# Modelling the cleanup of the North Pacific Garbage Patch based on 3 years of operational experience

**DOI:** 10.1038/s41598-026-40859-y

**Published:** 2026-03-20

**Authors:** Bruno Sainte-Rose, Laurent Lebreton, Yannick Pham, Arjen Tjallema, Christophe Maes

**Affiliations:** 1 The Ocean Cleanup, Rotterdam, The Netherlands; 2 The Modelling House, Raglan, New Zealand; 3 Université de Bretagne Occidentale, IRD, Brest, France

**Keywords:** Ocean sciences, Environmental sciences

## Abstract

Ending plastic pollution in the marine environment requires reducing the sources, preventing the plastics from entering the ocean, and removing the persistent legacy pollution accumulated in areas such as the North Pacific Garbage Patch (NPGP). This study assesses the feasibility and costs of collecting plastics accumulated in the NPGP during a 10 year-period, starting in 2027. The cleanup method consists of a fleet of U-shaped net systems towed behind two vessels funneling the plastic towards a retention zone which is periodically emptied. The background plastic mass concentration at the surface is based on a dispersal model calibrated on the results of field operations, and specifically on measurements performed during 72 collection periods which, between August 2021 and November 2024, led to the removal of a cumulated mass of 372,733 kg of plastics. The study evaluates the impact of the number of cleanup systems, the growth of the mass of plastics in the NPGP, the system’s retention efficiency, and how the systems are steered through the NPGP. Cost estimates are derived from vessel types, market rates, and past operational expenses. Results indicate that, after a decade, more than 80% of the total mass of plastics (> 1.5 cm) of the NPGP baseline can be eliminated, with up to 180 kt of plastics removed from an area covering an average of 1.6 M km^2^. This target could be reached at a cost of €1.8 billion in an optimal scenario, representing a negligible fraction (< 1%) of the value of the ecosystem services threatened by plastic pollution in this oceanic region for the next century.

**Supplementary Information:**

The online version contains supplementary material available at 10.1038/s41598-026-40859-y.

## Introduction

Plastic pollution in the marine environment is nearing planetary boundary threat conditions^1^. The steady increase of floating plastics in the marine environment is particularly concerning because of their ability to disperse in every part of the ocean surface while transporting invasive species^2^, endangering pristine habitats^3^ and affecting local communities that depend on a healthy marine environment^4^. Floating plastics threaten many species with the risk of ingestion or entanglement^5^ but also because of their ability to concentrate organic pollutants^6^ leading to a potential bio-accumulation along the food chain when ingested^7^. On a global scale, plastic pollution can disrupt zooplankton grazing, thereby affecting carbon cycling, especially in highly contaminated regions such as subtropical gyres and coastal areas of Southeast Asia and India^8,9^. When exposed to sunlight and oxygen, these plastics can slowly degrade by photo-oxidation into increasingly smaller pieces^10^, called microplastics. The continuous shedding of microplastics at sea from larger floating plastic objects can impact marine habitats not only at the sea surface of regions with high concentrations of microplastics^11^ but also in deeper layers of the ocean as smaller particles can be exported to depth by physical and biological processes^12^, likely already impacting species that may not have yet been discovered.

Global estimates of plastic inputs range from several hundred thousand to several million tonnes of plastic into the global ocean every year^13^. The most direct measure to mitigate this threat is to urgently reduce emissions by applying measures such as regulation on plastic production, that has been skyrocketing over the past two decades, or by developing environmentally friendly plastics or surrogate. Additionally, substantial investments by countries to improve waste management and drive a more circular economy of materials^14^ should further decrease emissions. The current pollution, known as legacy ocean plastics, will remain in the marine environment and continue to represent a threat if no action is taken to retrieve it^15^. If sources of plastic pollution were stopped, most floating plastic would return to shorelines with winds, waves, and tides^16–18^. Investing in systematic coastal cleanups would, therefore, be a cost-effective solution to reducing the amount of plastic in coastal environments^19^. However, areas in the global ocean, formed by rotating surface currents that act as traps for floating plastic pollution, are known to oceanographers as subtropical gyres^20^. There, no coastlines can capture this pollution, and floating plastics are believed to have accumulated for decades^16,17^. The North Pacific Ocean hosts one of the five subtropical oceanic gyres. It has been widely studied for the increasing quantities of floating plastics accumulating in these waters, creating what is known as the North Pacific Garbage Patch (NPGP). Surveys conducted in 2015 and 2016 concluded that between 45 and 129 thousand tonnes of floating plastics (> 0.5 mm) had accumulated over an area of 1.6 M km^2^^21^. Plastic pollution in the open ocean generates significant economic burdens across various sectors. A study estimated that marine plastic pollution leads to a 1–5% reduction in marine ecosystem services, translating to an annual loss of approximately $500 billion to $2.5 trillion globally based on 2011 USD ecosystem services valuations^22^. Additionally, it has been evaluated that the damage costs of marine litter on the marine economy in the Asia-Pacific Economic Cooperation (APEC) region increased from $1.26 billion in 2008 to $10.8 billion in 2015, with marine tourism bearing the majority of these costs^23^. Furthermore, a 2014 report by the United Nations Environment Programme^24^ estimated that plastic waste causes financial damage of $13 billion to marine ecosystems annually, impacting sectors such as tourism, fisheries, and shipping. Beyond the tourism and fishing sectors, often emphasized in coastal contexts, open ocean ecosystems also provide a wide range of ecosystem services, including carbon sequestration, which may be of equal importance in pelagic environments and coastal waters when considering their spatial spans^25^. Although open-ocean ecosystems are generally considered less valuable per unit area than coastal habitats (e.g., mangroves, coral reefs, seagrass meadows), their vast spatial extent—often spanning millions of square kilometers—renders their cumulative service value comparable to that of coastal environments. Hence, the cumulative ecological and economic importance of deep-sea and pelagic ecosystems is substantial^26–29^. In the specific case of the NPGP, plastic accumulation not only directly affects pelagic ecosystems but also extends to coastal environments such as the Hawaiian Islands, and particularly the Papahānaumokuākea Marine National Monument, the world’s largest marine protected area, while further intensifying cumulative impacts across marine food webs^30^. Collectively, these considerations underscore the relevance of cleanup and management initiatives in the NPGP.

The Ocean Cleanup, a not-for-profit project that develops and scales technology to intercept floating plastics before they reach the ocean and to retrieve legacy floating plastics from the subtropical gyres, has iteratively tested systems that collect floating plastics in the NPGP since 2018. The project has been particularly successful with the second and third generations of its systems (S002, a.k.a. Jenny, and S03, a.k.a. Josh) consisting of a long floating barrier (up to 2.2 km) with a retention zone towed by two slowly advancing vessels (< 2 kn). By the end of 2024, the organization had collected 504,229 kg of plastic waste (> 1.5 cm) from the NPGP (7,173 kg between 09/2018 and 10/2019 with S001 and S001B; 497,656 kg between 08/2021 and 11/2024 with S002 and S03). Using the learnings from the deployment of these systems and the resulting data collected during these tests, we propose in this study a roadmap to removing > 80% of floating plastics (> 0.5 cm) from the NPGP between 2027 and 2037 by scaling this technology taking into account uncertainties related to number of systems, retention efficiency, steering strategy and future growth of pollution in the region. We started by calibrating a plastic dispersal model with extracted quantities of floating plastics (> 1.5 cm) from 2021 to 2024 to assess future scenarios for the plastic mass in the NPGP for large meso-plastics (1.5–5 cm) and macro-plastics (> 5 cm). The evolution of the mass of small meso-plastics (0.5–1.5 cm) was derived from a mass balance model. Ten years of plastic dispersal hindcast were used for the background plastic in the cleanup model. Thirty-six scenarios were investigated, varying the four main parameters enumerated above. The reduction of the NPGP mass and mass of plastic removed were estimated for two size classes: plastics larger than the mesh size of the net (> 1.5 cm) and small meso-plastics (0.5–1.5 cm). The impact of an 80% source reduction starting halfway through the offshore cleanup operations was also investigated using a mass balance model. Finally, the cost of such cleanup operations could then be estimated.

## Methods

### Cleanup systems: extraction data and efficiency tests

**Fig. 1:**
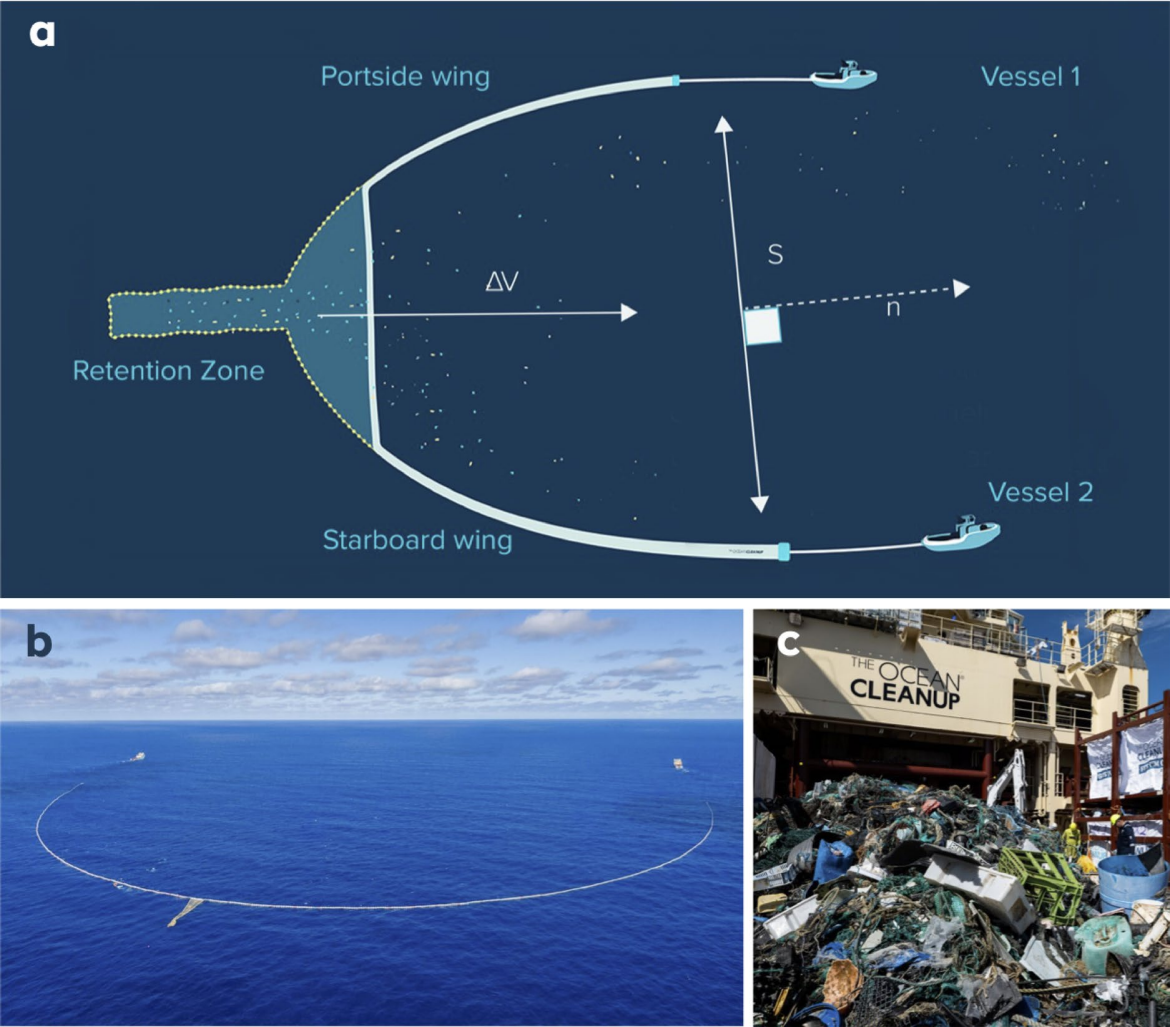
Sketch and images of The Ocean Cleanup plastic cleanup system and operation: (**a**) sketch of the Retention System (RS) and components along with parameters defining the swept area: speed through water vector Δ***V***, span S and vector normal to span **n**, (**b**) photograph of cleanup system S03 under tow configuration, image courtesy of *The Ocean Cleanup*, 2023. All rights reserved. (**c**) photograph of extraction #70 on deck before sorting, image courtesy of *The Ocean Cleanup*, 2023. All rights reserved.

Between August 2021 and November 2024, 23 missions occurred in the NPGP to test and scale a plastic cleanup system consisting of a U-shaped floating barrier (two wings) funnelling the plastic debris towards a netted contraption called the Retention Zone (RZ), with the overall system being called the Retention System (RS) as shown in Fig. 1 (panels a and b). The size of the square mesh employed for the net is between 1 cm and 1.6 cm, and further information on the system version characteristics (span, wing depth, RZ capacity, mesh size of the net) are given in Supplementary Table S1. During operations, continuous environmental management measures were implemented to monitor the surrounding marine ecosystem and to prevent and respond to potential incidents of significant bycatch^31^. These actions included measures built into the system design (design measures), measures based on how the system is operated (operational measures), and measures based on monitoring (monitoring measures). Design measures included high-frequency acoustic pingers and green LED lights installed to warn particular species of the system, and exit routes in case animals entered the RZ (large gaps in the bottom section and an active Retention Hatch System that could be triggered to allow animals to swim out of the RZ safely). Operational measures included slow towing speeds and avoiding areas with known concentrations of sea turtles. Finally, monitoring was performed by environmental observers that conducted above-water (scanning the surroundings of the vessels and system with the unaided eye and binoculars, during transit and towing) and underwater (continuous video streaming of cameras located inside the RZ during towing) observations to detect potential protected species and eventually trigger a response to avoid negative interactions. Periodical extractions were performed to remove the catch from the RZ’s extraction section and offload them on the deck of one of the two ships utilized to tow the RS, as depicted in Fig. 1 (panel c). Once the catch was safely onboard, plastic debris was mechanically and manually sorted to separate the bigger items, which measured more than 5 cm. This debris was then divided between fibrous (i.e., fishing nets and ropes) and rigid and subsequently weighed. The remaining items (1.5–5 cm) were also weighed but not sorted. Throughout the operations, the speed through water $\:{\Delta\:}\boldsymbol{V}$ (in m/s) and the system’s span $\:S\:$ (in m) are monitored, respectively, with the ships’ Doppler Velocity Logger (DVL) and GPS trackers mounted at several locations along the wings to monitor their shapes. The instantaneous swept area is then given by making the scalar product $\:{\Delta\:}\boldsymbol{V}.S\boldsymbol{n},\:$ where $\:\boldsymbol{n}$ is the unit vector normal to the span line, as sketched in Fig. 1 (panel a), and summed over the different collection periods, which correspond to the time between two extractions when the RS’s span is opened and the extraction section of the RZ is installed and closed. The average extracted plastic surface mass concentration (in kg/km^2^) for plastics (> 1.5 cm) is obtained by dividing the extracted mass by the total swept area, see Supplementary Methods S1. The extracted dry mass of plastics and surface coverage area for individual collection periods are provided in Supplementary Table S2. This table also provides the total weight of bycatch (primarily bony fish, sharks, and invertebrates) caught by the system or associated with/trapped in plastic. Supplementary Fig. S3 depicts the boxplot of the ratio between extracted bycatch mass and plastic mass. This ratio is in general very low (minimum: 0.03%, p75: 0.25%, p50: 0.58%, p25: 0.91% and maximum: 10.5%) and shows that the cleanup operations caught significantly more plastic than marine species.

A series of tests has been performed to measure the RS’s efficiency. This efficiency is defined as the mass of plastics extracted from the system relative to the mass that enters the “span line” of the RS (i.e., the imaginary line between the two endpoints of the U-shaped system). During efficiency tests, labelled plastics were released in front of the system’s span line. Groups of different types and sizes of plastic were deployed at several locations along the span line to assess the whole system’s performance, as further described in Supplementary Methods S2. The tested objects were followed by the Fast Rescue Craft of one of the vessels and multi-rotor Unmanned Aerial Vehicles for observation. After extraction, the collected plastics were sorted, and the labelled items were retrieved and separated. The retention efficiency was determined as the ratio of retrieved to released labelled items (for different types and sizes).

### Description of the NPGP: baseline and pollution growth rates

**Fig. 2:**
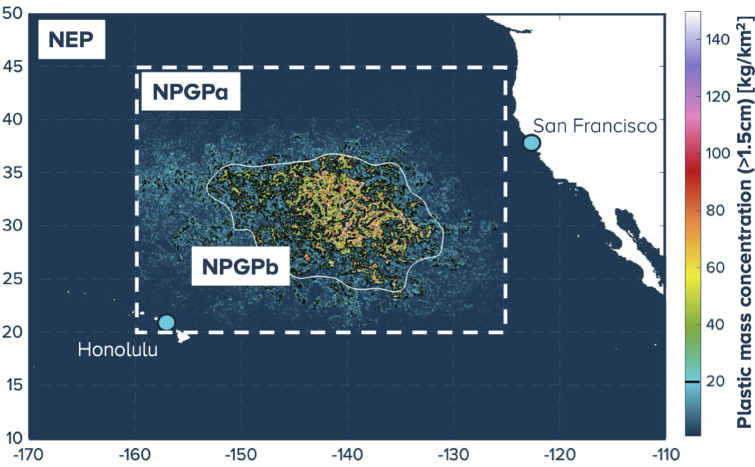
Definition of the boundaries of the areas of interest: NEP and NPGPa (dashed white rectangle), NPGPb (black iso-lines), the white iso-line delimits a smoothed NPGP used in the steering strategy scenarios introduced in the following paragraph; colors represent the indicative accumulation of floating plastics (> 1.5 cm) simulated by the dispersal model for July 2024.

The accumulation of floating plastic debris in the North Pacific subtropical gyre was simulated using a global Lagrangian dispersal model. In short, 1.2 M numerical particles were released from global plastic pollution source locations along the coastline^21^ and advected by sea surface currents using the ADVECT software^32,33^. For this exercise, we used 31 years of ocean circulation data from the global GLORYSv12 reanalysis^34^, from January 1993 to November 2024. Three regions were defined as depicted in Fig. 2: the North East Pacific study region (NEP) defined between 10 °N-50 °N and 170 °W-110 °W, the NPGP area (NPGPa) defined between 20^o^N-45^o^N and 160^o^W-125^o^W, and the NPGPb defined by the area containing 75% of the modelled mass in NPGPa which covers in average around 1.6 M km^2^^21,35^.

We used the measurements taken during 72 collection periods (out of 103) corresponding to 372,733 kg (out of 497,656 kg). 31 collection periods were discarded for numerous reasons (GPS / DVL malfunctions, damages on the net, or wings in a degraded configuration: twisted wings, deflated buoyancy units, apparition of holes on the wing screen, emergency releases), a breakdown of the reason why specific collection periods were discarded is given in Supplementary Table S3. In addition, the measured wind conditions for the kept collection periods were statistically worse than the discarded ones as detailed in Supplementary Methods S4 and Supplementary Table S4. For each system configuration (S002-S002A / S002B / S002C / S03), the encountered Lagrangian particle concentrations mapped on a 0.08°×0.08° resolution grid of the NEP area were first corrected by wind-dependent retention efficiencies derived from the tests detailed in Supplementary Methods S2. Note that S002 and S002A are grouped because there were no modifications on the wings, only a different Retention Zone, which should have a negligible impact, see Supplementary Table S1 for more information on the system characteristics. The mass of individual Lagrangian particles was then estimated from a linear regression, as detailed in Supplementary Methods S3. Finally, the total mass inside the NPGPb area can be evaluated through a similar calibration exercise as performed by^21^, based on an extensive sampling effort across size classes, with an estimated 79 (low = 45 and high = 129) thousand tonnes of floating plastics (> 0.5 mm) with 68 (low = 38 and high = 108) thousand tonnes of plastics (> 1.5 cm) over an area of 1.6 M km^2^ as of summer 2015. Two annual growth rate (a.g.r.) scenarios (1% and 3%) of the mass in the NPGP were considered in agreement with previous works^16,17^. In the results section, the credibility of the mid scenario is assessed against the NPGPb masses obtained after calibrating the current model.

### Simulation of a fleet of cleanup systems

First, to reproduce the background plastic surface mass concentration encountered by the cleanup systems during the 10 years of cleanup operations starting in on January 1st 2027, 10 years of dispersal model hindcast were selected (2009–2019). To accurately model the cleanup effect across the operations’ timeframe, it is crucial to keep a Lagrangian framework to consider the evolution of the mass of the cleaned particles and the appropriate growth of their number to reproduce the desired a.g.r. of the NPGP. Second, to simulate the cleanup operation of plastics (> 1.5 cm), N cleanup systems working similarly to S03 were considered for the 2027–2037 period, operating with a span of 1800 m (average simulated span for a 2500 m long system) and 0.75 m/s speed through water $\:{\Delta\:}V$. The other governing parameters of the cleanup system’s operation are retention efficiency and uptime, which was fixed at 80%, corresponding to an achievable target based on the median uptime experienced during the 2021–2024 operations (70% see Supplementary Table S5 for experienced uptimes), considering continuous rotations and year-long operations. The plastic mass concentration at the surface in the NPGP is highly heterogeneous spatially, as evidenced for microplastics^36^; in fact, it is driven by the complex interaction between oceanic eddies, creating accumulation of passive floating material or biological tracers at all geophysical scales (oceanic gyres, mesoscale and submesoscales)^37,38^, sometimes yielding persistent convergence fronts which could be evidenced by drifters^39^. Therefore, we anticipate the cleanup fleet’s steering strategy significantly impacting the recovered plastic mass. In this study, three steering strategies were introduced: (i) random steering inside the NPGP, (ii) a hotspot hunting strategy (HSH), which consists of short-sightedly looking for the closest area of accumulations on a 3-daily basis, and finally, (iii) an optimal steering scenario that picks the most rewarding tracks over 7-days using a dynamic programming algorithm^40^; the cleanup model and steering strategies are further explained in Supplementary Methods S4 and S5.

We focused on the influence of four parameters: annual NPGP mass growth rate, system retention efficiency, number of systems, and steering strategies. The table gives the values used for the 36 scenarios (two a.g.r., three steering strategies, three system numbers, and two retention efficiencies).

**Table 1:** Parameters used in the NPGP cleanup simulations.

Parameters	Unit	Values
a.g.r.	[%]	1–3
Steering strategy	–	RND - HSH - OPT
Number of systems	[#]	10-15-20
Retention efficiency	[%]	40–70
Span	[m]	1800
Uptime	[%]	80
Speed through water	[m/s]	0.75

### NPGP mass balance model

The performance of the NPGP cleanup is evaluated for plastics > 0.5 cm; consequently, a mass balance approach was proposed to model the evolution of the mass concentration of small meso-plastics (0.5–1.5 cm) since plastic collected by the cleanup operation and used to calibrate the model had a minimum size of 1.5 cm, corresponding to the mesh size of the net of the system. We hypothesized that the net inflow of plastics of all size classes evolves at a 4% a.g.r. corresponding to the global growth in plastic production^16^. The plastics are grouped into two size classes: small meso-plastics and larger plastics (> 1.5 cm). To model the degradation rate between these two size classes, we used a fragmentation approach^41^ with parameters ($\:\lambda\:\:$= 0.0005 to 0.05 /y and *p* = 0.4), which are within the ranges investigated in a previous study^42^. This gives annual degradation rates between 0.4% and 1.2% for plastics (>1.5 cm) to (0.5–1.5 cm) and between 2% and 6% for plastics (0.5–1.5 cm) into microplastics (<0.5 cm). Further details can be found in Supplementary Methods S7. We can subsequently assess the impact of reducing the sources by acting on the net inflows. Therefore, an 80% inflow reduction after 5 years of operations was also investigated for the scenarios in Table 1.

### Cost estimation

The total cost of cleanup was estimated from the number of cleanup systems in operation and the duration of the cleanup. For this purpose, the cleanup operation and all its steps were simulated, resulting in the number of ships required to meet the cleanup objective in a set time frame. The type of ships, associated costs and capabilities, and the operational model are key inputs that drive the total cost of cleanup. The operation was modelled using the ship type and operational setup that is expected to be most cost-effective. In this setup, ships of the Platform Support Vessel (PSV) type are deployed, with sufficient towing capability for towing the RS at the desired speed and span and sufficient space to perform the complete operation. More than two ships per RS are in operation, such that every time a vessel is due for a port call, it is relieved by one of the additional vessels in the fleet. The RS can then proceed with cleanup operations without significant disruption. The total number of vessels required to perform this operation depends on the trip length of each vessel and is an output of the simulations performed. When in operation, each ship is to be capable of carrying out the different steps of the cleanup operation: (1) transport of RS(s) to and from the NPGP when due for inspection, repair, and maintenance, (2) deployment and recovery of RS(s), (3) plastic extraction from the RS, (4) transport of extracted plastics to shore, and (5) towing of the RS. The latter is the part of the operation where the plastics are collected. The simulation computes the time each vessel and each RS spend on the different activities, with associated fuel consumption, resulting in an average uptime percentage for the RSs. The operation maximizes this uptime to achieve the best possible cleanup performance. For the cost estimate, a market scan was performed for the type of vessels foreseen to be deployed in operation, resulting in market-conform cost and fuel consumption figures for the vessels, crew, and operations. These have been taken into the simulation and are corrected for inflation throughout the cleanup. The parameters used to model the costs are given in Supplementary Table S6. The current estimates are based on cost information received as of November 2024.

## Results

### Testing the system efficiency and NPGP model calibration

**Fig. 3:**
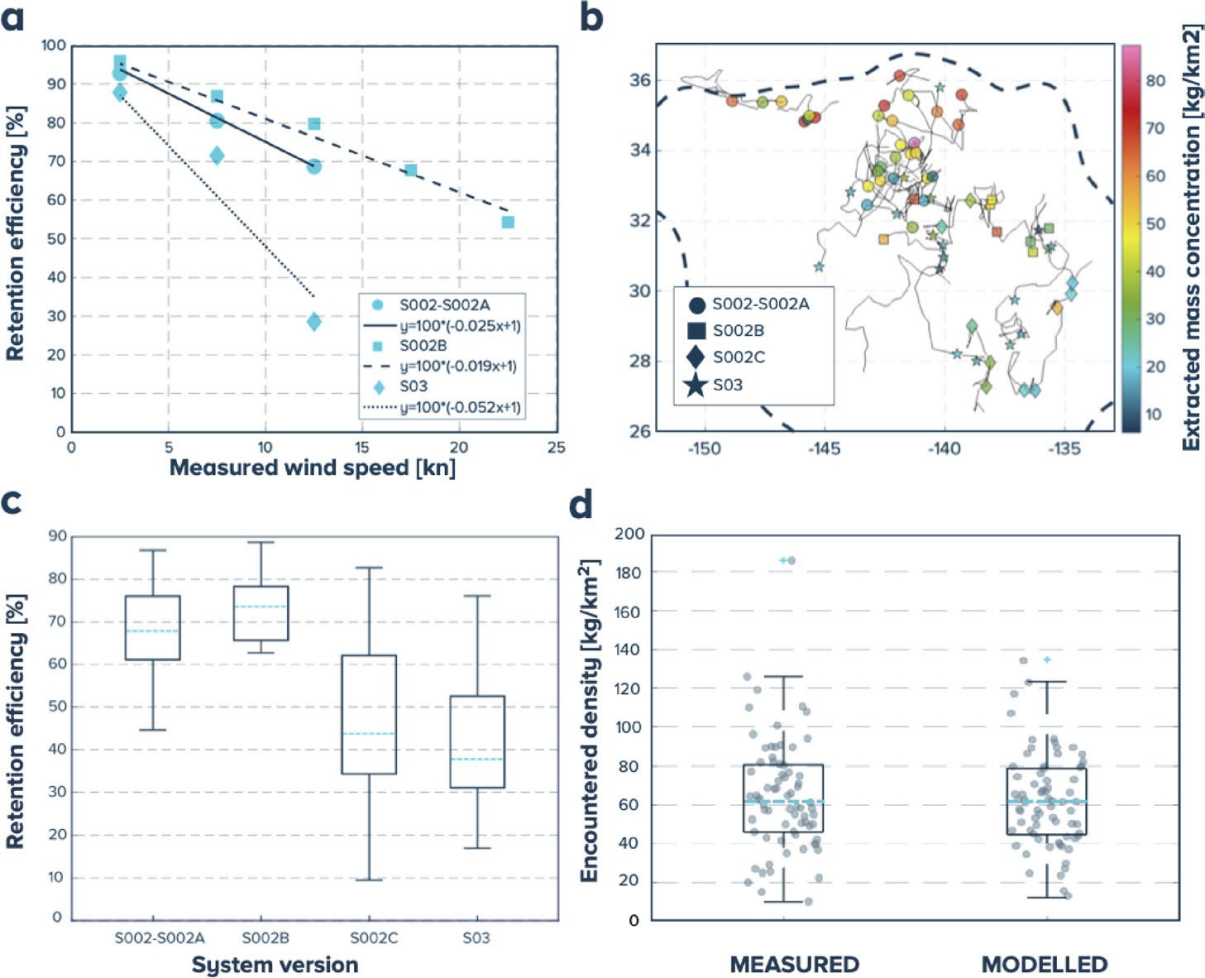
Extractions and efficiency tests, and model calibration results: (**a**) measured retention efficiency results (%) binned by wind speed (in knots) and linear law for the three versions of the system, (**b**) tracks of the 72 collection periods considered with the corresponding extracted mass concentration (kg/km^2^), (**c**) boxplots of the estimated retention efficiency (%) for the different system versions, (**d**) boxplots of the encountered concentrations estimated from the measurements (MEAS.) and calibrated model (MOD.), in the background, all 72 values for each case.

To estimate the retention efficiency of the cleanup systems for the different versions and under different environmental conditions, 21 retention efficiency tests were performed for S002-S002A, S002B, and S03. Linear relations between efficiency and measured wind speed were derived from these tests, as shown in Fig. 3 (panel a). Conservatively, we assumed that S002C follows the same linear relationship as S03. More details on the outcome of those tests are given in Supplementary Fig. S2.

The tracks and corresponding extracted mass concentrations for the 72 collection periods are depicted in Fig. 3 (panel b). For each collection period, the modelled retention efficiency was computed based on the encountered wind conditions; boxplots of this retention efficiency are given for the four system versions in Fig. 3 (panel c). The average retention efficiency for the different system configurations is shown in Table 2. These values are in line with the hypothesis taken for the retention efficiencies of the 10 years simulations (40% corresponding to averaged S03 performance and 70% corresponding to realistic performance improvements which are currently being investigated experimentally to optimize the design parameters such as mesh size, screen depth and lines arrangement) given that the conditions encountered by the cleanup systems were statistically worse for S002-S002A and S002B (which experienced the most winter conditions), equal for S03 and milder for S003C, than the multi-year modelled wind conditions in the center of the NPGP as further described in Supplementary Methods S4. The retention efficiencies are then used to derive the average plastic mass concentration encountered by the RS during each collection periods. The mass concentrations encountered in the calibrated dispersal model is compared to the measured encountered concentrations and are in good agreement in terms of statistics (median: 61.8 kg/km^2^ resp. 61.1 kg/km^2^, p25: 46 kg/km^2^ resp. 44.6 kg/km^2^, p75: 80.7 kg/km^2^ resp. 78.8 kg/km^2^ for measurements resp. model) with a p-value of 0.96 for the Kolmogorov-Smirnov test and as further evidenced in the boxplots Fig. 3 (panel d), see also Supplementary Fig. S4.

The total mass inside NPGPb is calculated for the different system configurations in Table 2. The difference between the mid-scenario with 1% resp. 3% a.g.r. and the calibrated model results is between − 0.6% resp. -14.8% and − 8.3% resp. -21.8%.

**Table 2:** Comparison between NPGPb plastic mass (> 1.5 cm) estimates from collected mass and plastic dispersal model vs. mid-point estimate^19^ and modelled averaged retention efficiency.

RS version	#	Modelled NPGP mass [t]	Modelled efficiency (avg) [%]	Mid (1% agr) [t]	Error [%]	Mid (3% agr) [t]	Error [%]
S002-S002A	32	66,277	68	72,244	-8.3	82,198	-19.4
S002B	8	70,766	73	72,845	-2.9	84,248	-16.0
S002C	10	72,753	46	73,178	-0.6	85,397	-14.8
S03	22	68,376	40	73,757	-7.3	87,419	-21.8

### Baseline scenario for plastics (0.5–1.5 cm) and (> 1.5 cm)

**Fig. 4:**
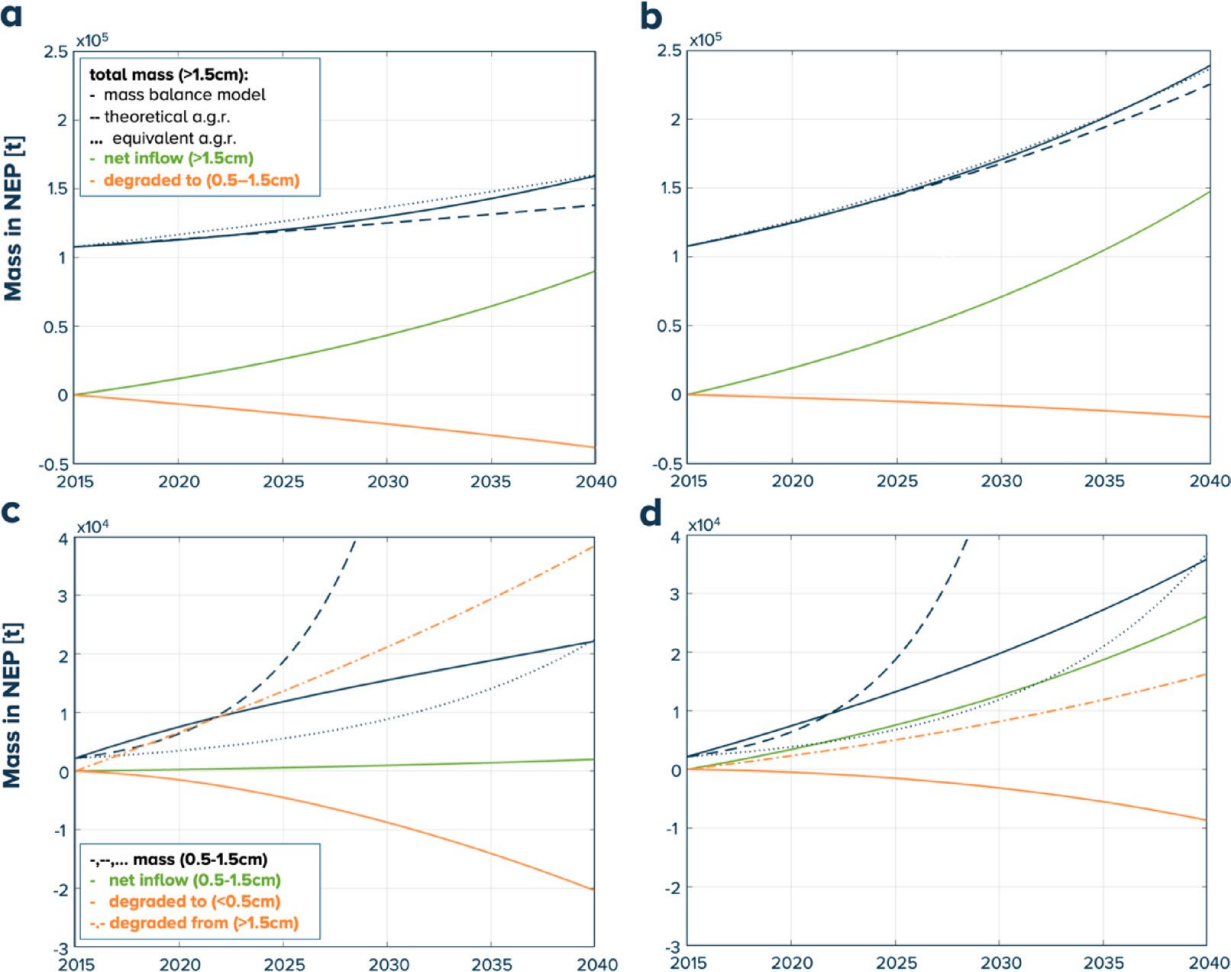
Evolution of the total mass inside NEP, net inflows, degraded mass of the plastics: (**a**, **b**) plastics (> 1.5 cm): and (0.5–1.5 cm): (**c**, **d**). Baseline scenario with mass balance and 1% a.g.r. ; (**b**, **d**) and 3% a.g.r.: (**a**, **c**).

To model the baseline scenario of all size classes (> 0.5 cm), the starting net inflow in 2015 for the different size classes is determined to match 1% or 3% average a.g.r. of (> 1.5 cm) plastics and 24% average a.g.r. of (0.5–1.5 cm) plastics over the 2015–2022 period in alignment with historical measurements^35^ and a previous holistic global simulation^16^. This approach yields a starting net inflow of 2,195 t/y resp. 3,757 t /y of (> 1.5 cm) plastics and 80 t/y resp. 673 t/y of (0.5–1.5 cm) plastics into the NEP for the 1% resp. 3% scenario. This also allows us to assess the impact of reducing the sources by acting on the net inflows. Figure 4 shows the different contributions in the baseline scenario.

### Impact of the cleanup scenarios on the NPGP content

**Fig. 5:**
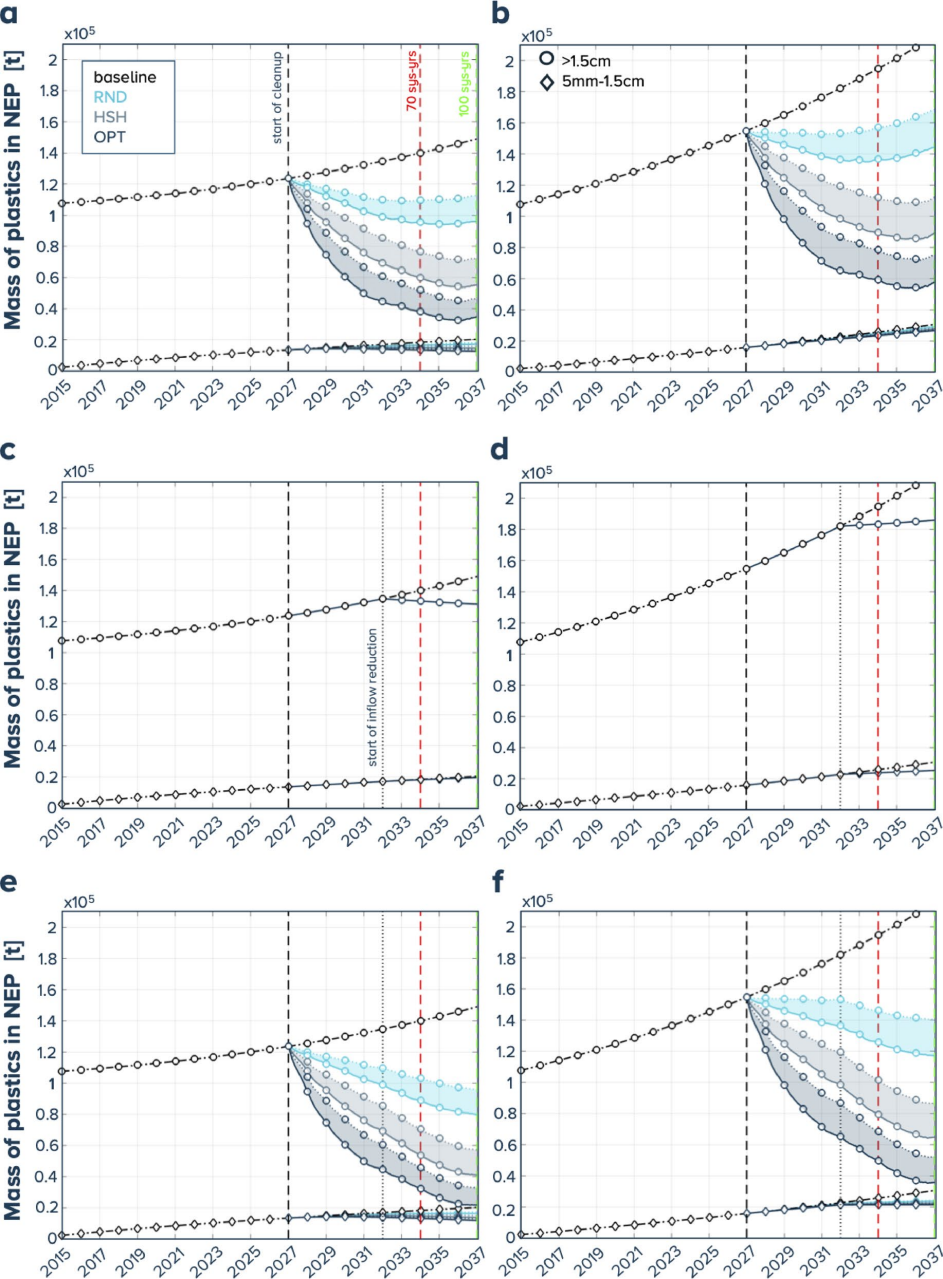
Evolution of the mass inside the NEP region (in tonnes) for the different steering strategies: RND for random steering, HSH for hotspot-hunting and OPT for optimized (corresponding colors given in panel **a**) and the two size classes (corresponding symbols given in panel **b**) for 10 cleanup systems starting in 2015 (solid lines: retention efficiency 70%, dotted lines retention efficiency 40%): (**a**, **c**, **e**) resp. (**b**, **d**, **f**) correspond to 1% resp. 3% a.g.r. of (> 1.5 cm) plastics and from top to bottom row: offshore cleanup only (**a**, **b**), inflow reduction only (**c**, **d**), the combination of both (**e**, **f**); vertical dashed lines, start of cleanup and / or inflow reduction, 70, 100 system-years.

During the 10 years of simulated cleanup, our model suggests a mass reduction of plastics (> 0.5 cm) (compared to the NEP baseline) between 20 and 50% for the RND steering strategy, 43 and 70% for the HSH strategy, and 58 and 79% for the OPT steering strategy without reduction of the net inflow; 10–14% when the net inflow alone is decreased by 80%; when combined with offshore cleanup this yields reductions between 33 and 60% for the RND steering strategy, 55 and 78% for the HSH strategy and 70 and 86% for the OPT steering strategy. The detailed results of the offshore cleanup performance after 10 years are given for all 36 cases in Supplementary Table S7. To give an example, the temporal evolutions of the mass of plastics in the NEP for the catchable size classes (> 1.5 cm) and small meso-plastics (0.5–1.5 cm) for the scenarios with 10 cleanup systems are plotted in Fig. 5 for 1% and 3% a.g.r. scenarios between 2015 and 2037. Similar figures for 15 and 20 systems are provided in Supplementary Fig. S10 and Supplementary Fig. S11.

**Fig. 6:**
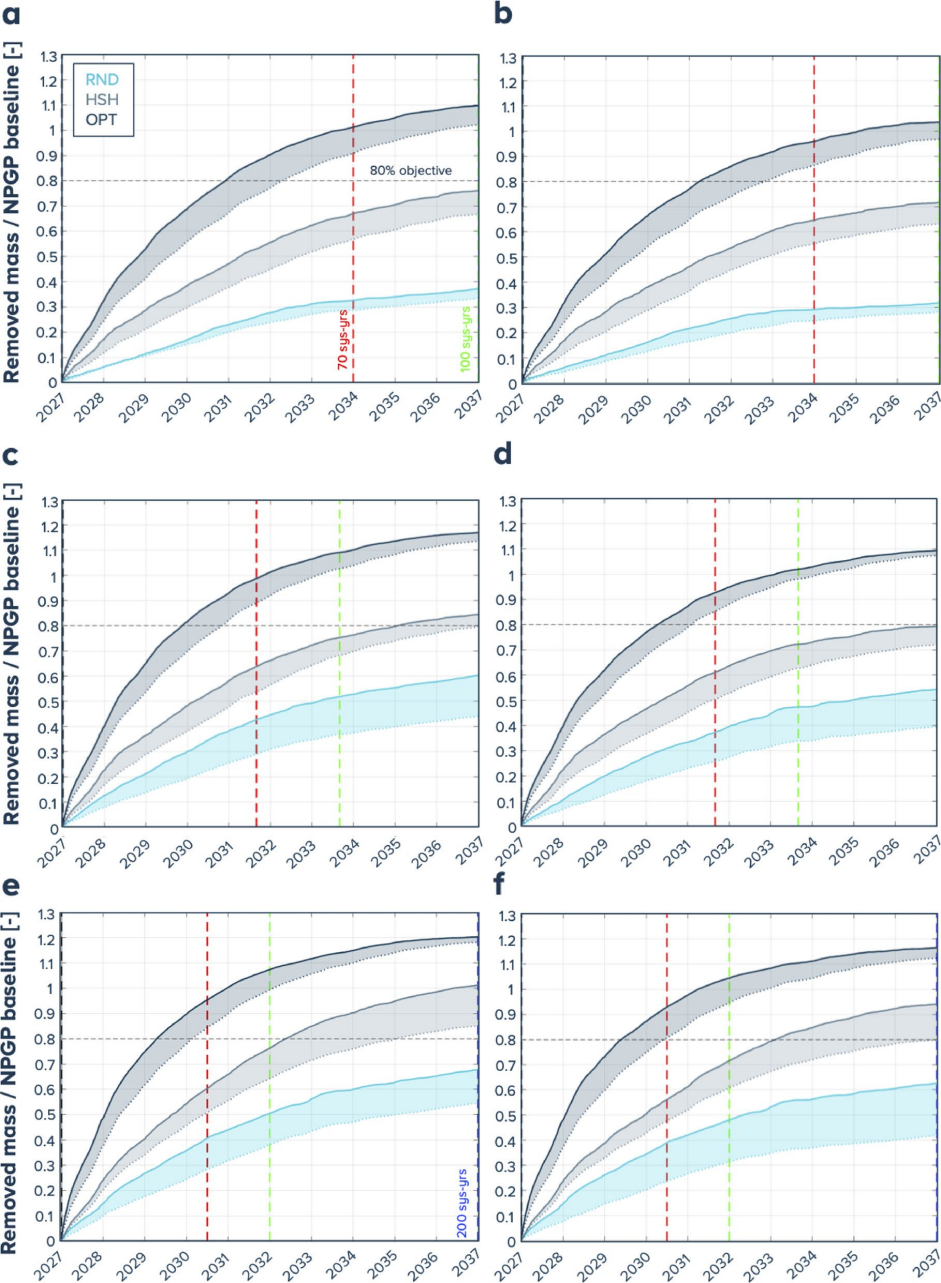
Evolution of the ratio between total removed plastic mass (> 1.5 cm) and mass inside the NPGPb baseline for the different steering strategies: RND for random steering, HSH for hotspot-hunting and OPT for optimized (corresponding colors given at the top of panel **a**) (solid lines: retention efficiency 70%, dotted lines retention efficiency 40%). (**a**, **c**, **e**) resp. (**b**, **d**, **f**) correspond to 1% resp. 3% a.g.r. of (> 1.5 cm) plastics and from top row to bottom row: 10 systems - (**a**, **b**), 15 systems - (**c**, **d**), 20 systems - (**e**, **f**); vertical dotted lines 70, 100, 200 system-years.

Focusing on offshore cleanup only, the evolution of the ratio between the total mass removed by the cleanup systems and the mass of plastics (> 0.5 cm) in the NPGPb baseline is shown in Fig. 6 for 10, 15, and 20 systems. We set a target objective of removing more than 80% of the mass of plastics (> 0.5 cm) in the NPGPb. Within the 10 years simulated, this objective was never reached for the RND steering scenarios. For the HSH scenarios and 1% a.g.r. and 70% retention efficiency, the model suggests the objective could be reached within 9 years for 15 systems and 8 years for 20 systems. For the OPT steering strategy, this objective was reached in our model for all cases, with the earliest being 40 system-years for 1% a.g.r. and 70% retention efficiency with 10 systems (4 actual years) and latest after 70 system-years years for 3% a.g.r. efficiency and 20 systems (3.5 actual years). Note that this ratio can become greater than 1 because of the circulation between the regions (NPGPb, NPGPa, NEP) where particles that belonged to NPGPb can be cleaned and then leave this region, being replaced by new ones, therefore not participating in the total mass reduction in the initial region.

**Fig. 7:**
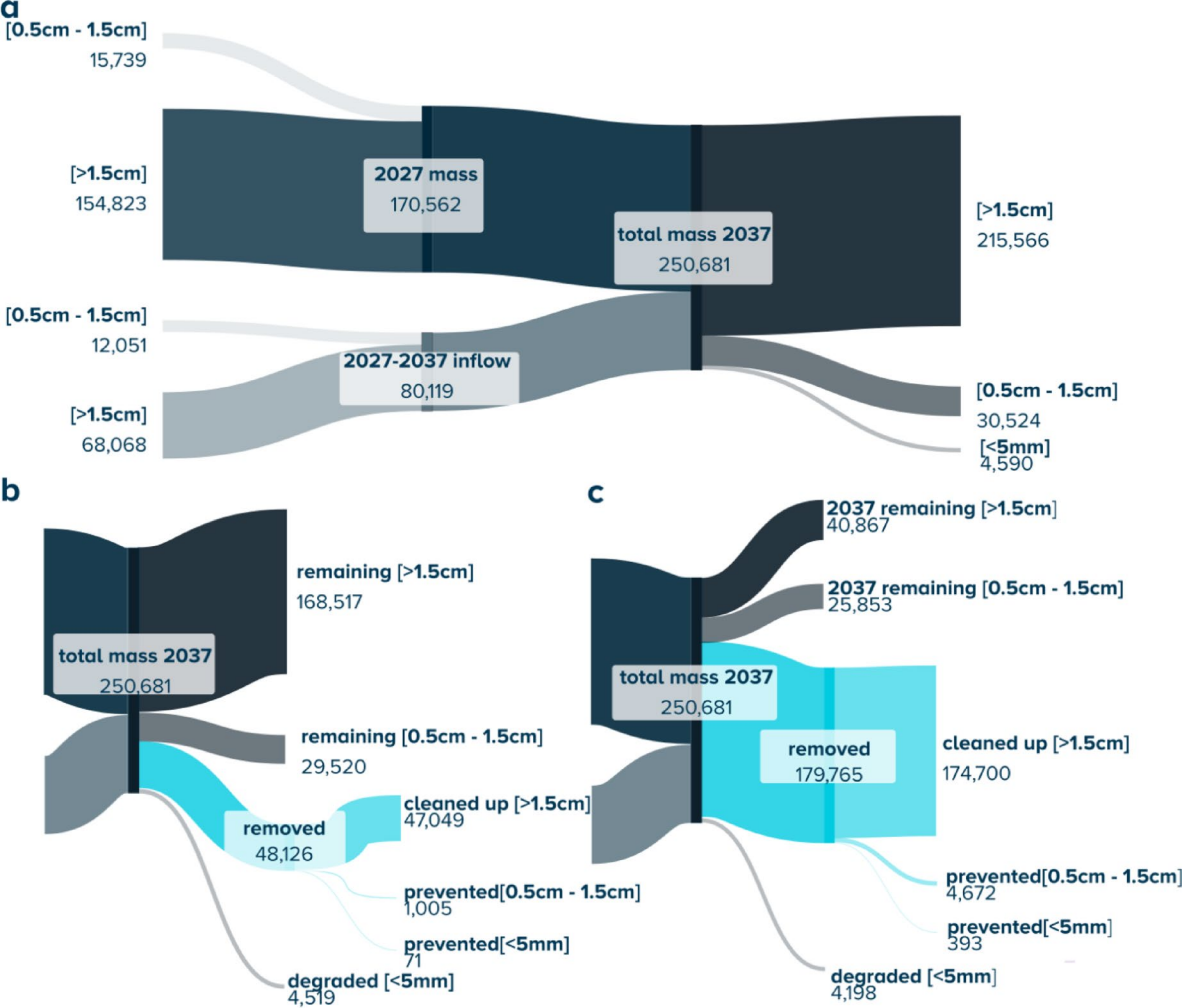
Sankey diagrams showing the evolution of the total mass in NEP for the different size classes (in tonnes): (**a**) baseline scenario for 3% a.g.r., (**b**) worst case (10 systems, random steering strategy and 40% retention efficiency) and (**c**) best case (20 systems, optimized steering strategy and 70% retention efficiency) for 3% a.g.r.

The fate of the plastics of the different size classes, namely (> 1.5 cm), (0.5–1.5 cm), and (< 0.5 cm) with or without offshore cleanup in the NEP region between 2027 and 2037 is depicted using a Sankey diagram (for 3% a.g.r.) in Fig. 7; the same diagram is produced for the 1% a.g.r in Supplementary Fig. S12. The impact of the cleanup on the different size classes is further highlighted: while the cleanup can have a significant impact on the evolution of the plastics larger than the mesh size of the.

net (> 1.5 cm), reducing from 149 kt – 216 kt (1–3% a.g.r.) in the baseline to between 113 kt − 169kt resp. 11 kt – 41 kt for the worst resp. the best scenario, it has a relatively low impact on the smaller size.

class; for the small meso-plastics (0.5–1.5 cm), it is reduced from 20 to 31 kt (1–3%) in the baseline to between 19 kt – 29 kt and 16 kt – 25 kt for the worst resp. best scenario.

### Impact of steering strategies on encountered plastic mass concentrations

**Fig. 8:**
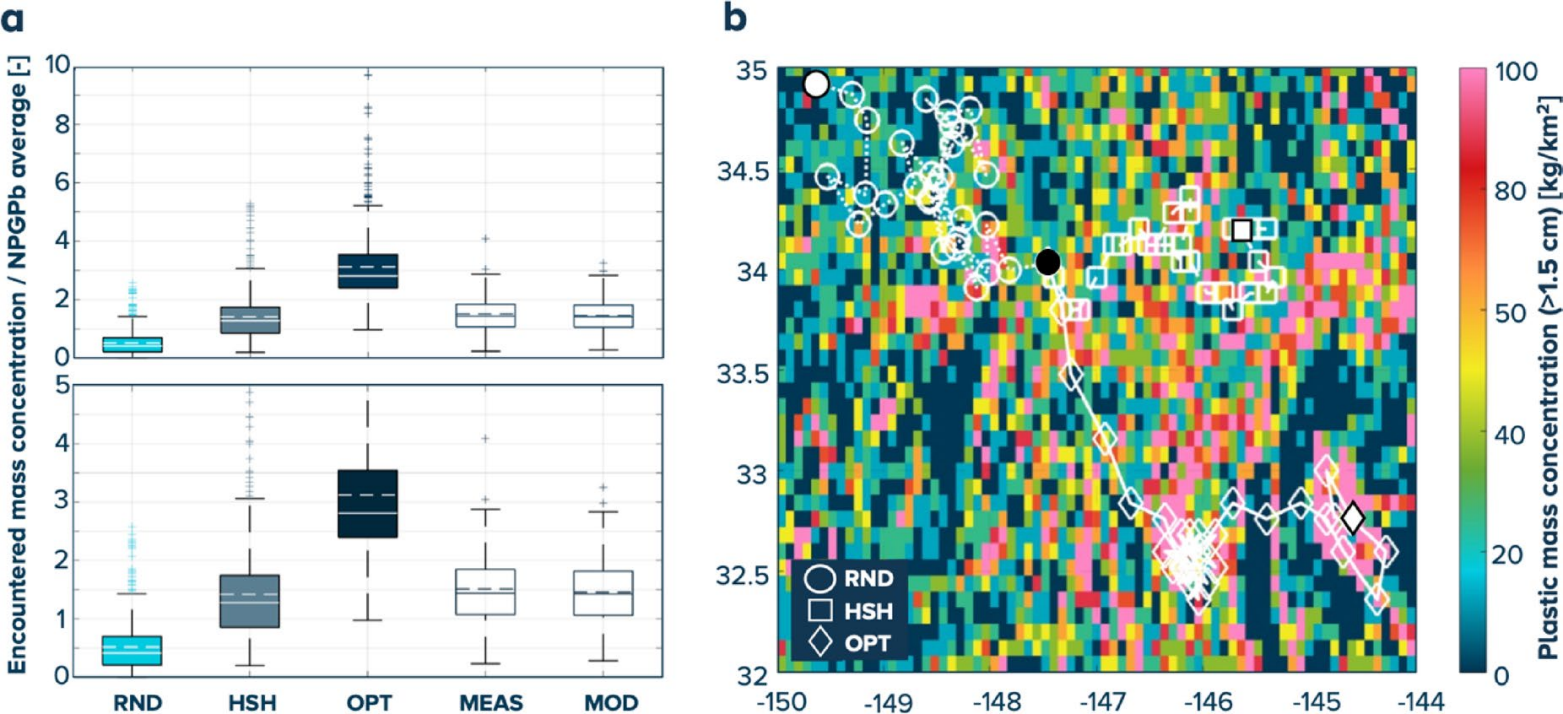
Effects of the steering strategy on the encountered mass concentrations: (**a**) boxplots of mass concentration encountered by the cleanup systems normalized by the average mass concentration in NPGPb (dimensionless ratio) for the three steering strategies (RND for random steering, HSH for hotspot-hunting and OPT for optimized) and experienced during our operations either measured from the extractions (with the retention efficiency correction) (MEAS) or modelled (MOD), the dashed horizontal lines in the boxes correspond to the average and the solid ones to the median; (**b**) example of cleanup systems tracks for the different steering strategies (legend in the figure, the starting location in black and ending locations in white).

As evidenced in Figs. 5 and 6, the main performance driver of the cleanup is the steering strategy. The ratio between the plastic mass concentrations encountered by the cleanup systems, normalized by the averaged concentration in NPGPb, was analyzed to evaluate the steering strategies. Figure 8 (panel a) shows boxplots of this quantity for the three steering strategies (RND, HSH, and OPT) and the measured (wind-corrected) extracted (MEAS) and modelled (MOD) mass concentrations. This figure shows that the plastic concentrations encountered during previous operations were comparable to those expected from the HSH steering strategy. An example of tracks obtained for the different steering strategies is plotted over the plastic mass concentration maps in panel b. In this figure, it is qualitatively highlighted that, given the heterogeneous nature of the NPGP, some level of optimization can easily yield a premium in the plastic mass concentrations encountered compared to a random motion.

### Cost estimations

**Fig. 9:**
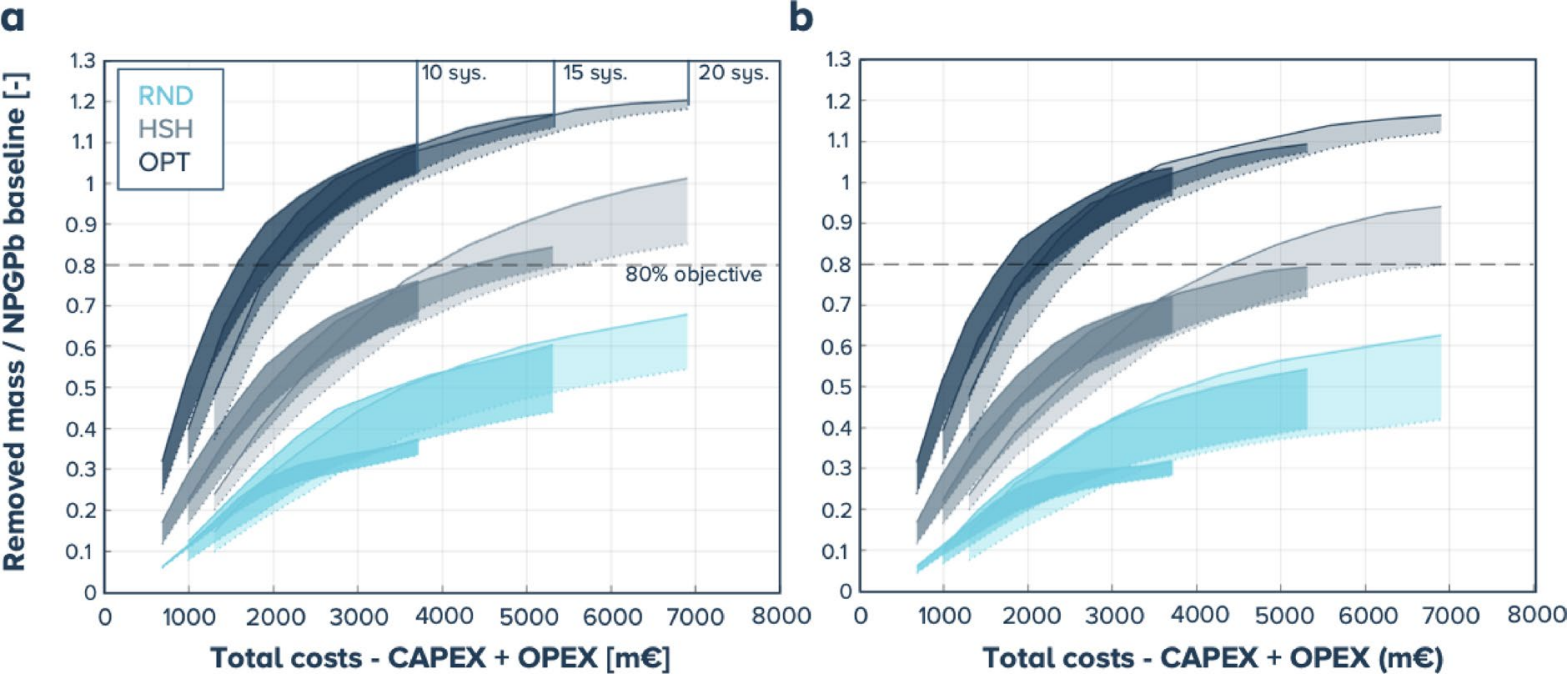
Removed mass / NPGPb baseline as a function of the total costs, including CAPEX and OPEX (in m€): (**a**) 1% a.g.r. and (**b**) 3% a.g.r. (solid lines: retention efficiency 70%, dashed lines retention efficiency 40%), the meaning of the colors is given in the inset on panel **b**: RND for random steering, HSH for hotspot-hunting and OPT for optimized.

For each year of operation, the average cleanup cost per system is determined, as detailed in Supplementary Table S8. The total cost of operations is plotted against the ratio between mass removed and total mass in the NPGPb baseline in Fig. 9 for 1% (panel a) and 3% a.g.r. (panel b). This plot shows that for 10, 15, or 20 systems and the OPT steering strategy, the 80% objective can be reached within 10 years and between 1.7 and 2.6 B€ resp. 1.8 and 2.7 B€ for 1% resp. 3% a.g.r. For the HSH steering strategy, the objective is only achieved for the 1% a.g.r, yielding costs between 3.8 and 6.9B€.

The cost of the cleanup obtained by scaling the performance of the systems that have been trialed between 2021 and 2024 are compared to the modelled cleanup scenarios as detailed in Supplementary Methods S8. Supplementary Figure S13 shows that the cost would vary between 45B€ when scaling S002A performance to less than 4B€ for future systems with an optimized steering strategy.

## Discussion

The total mass of plastics (> 1.5 cm, the mesh size of the net) at the surface of the NPGP, calculated with our dispersal model and calibrated with the extraction data, is in the same range as the mid-scenario baseline with 1 to 3% a.g.r. from 2015 to 2022^21,35^. Table 2 shows that, depending on the system version, negative errors range from − 0.6% to − 21.2% (varying with the a.g.r. scenario). These numbers can have two explanations: the mass in the NPGP could be lower than the mid-scenario (either because of a smaller mass in 2015 or a smaller a.g.r.), or the retention efficiency could likely be overestimated since the plastic pieces used in the retention efficiency tests were taken from previously collected plastics, introducing a bias that is hard to quantify. Additionally, it is worth noting that apart from S002-S002A, the worse conditions covered by the retention tests don’t go above the median conditions in the NPGP (12.5 knots) hence challenging the validity of such an approach for higher winds. Continuous underwater observations of underflow were also conducted at specific locations within the system to refine the understanding of retention efficiency further. However, the ability to segment the images and derive plastic motions was at too early a stage, and the coverage was too scarce to be conclusive. More systematic observations combined with physical and numerical modeling of plastic-system interaction should tend to improve those estimations in the future.

Looking at the mass balance, the evolution of the small meso-plastics size class (0.5–1.5 cm) is governed by degradation from larger plastics and by degradation into microplastics. These values, obtained using a fragmentation formula, could be debated, especially for larger and more complex plastics, including ghost nets, whose mass can increase over time through aggregation and move to a higher size class. The hypothesis on the evolution of the net inflow (4% for both size classes) can also be challenged. However, since no specific measurements are available to estimate this parameter accurately, the net inflow to the NPGP is assumed to follow the commonly accepted inflow of plastics into the global ocean as if the system was in a steady state. Yet it could be more complicated in reality when considering delays between emission from sources and accumulation to the offshore ocean. Plastic concentration sampling outside accumulation areas and better quantification of meso- and macro-plastic fragmentation could further refine those assumptions.

From the simulated cleanup scenarios, the main performance driver of the cleanup is the steering strategy, going from around 30% of the plastics (> 0.5 cm) in the NPGP cleaned using a random strategy to above 80% with an optimized steering, which requires an accurate knowledge of the plastic mass concentration forecasts. However, its impact should also depend on the transport model (combining ocean circulation, wind, and wave-induced drifts). A resulting dispersal model exhibiting a higher heterogeneity should yield a higher premium with dedicated steering than a more diffusive model. That is why improving the modeling of the transport mechanisms and the horizontal mixing induced by the background circulation is paramount to better evaluating the cleanup performance.

When considering NPGP cleanup only, the ratio between the mass remaining in the NEP reaches, at best, a plateau and sometimes rises again at the end of the ten years (as visible in most cases shown in the upper part of Fig. 5); this is when the amount of removed plastics every year is below than the net inflow of plastics. In particular, the cleanup does not act on plastics (< 1.5 cm) other than removing those that would otherwise degrade, which is relatively low given the expected low degradation rates in the ocean. Therefore, the only way to avoid this plateau and reach ratios below 20% of the baseline scenario (see also Supplementary Figs. S10 and S11) is naturally to reduce the inflow in parallel (as shown at the bottom of Fig. 5). Thus, to achieve sustained impact and maintain low plastic concentrations, we require a combination of cleanup and inflow reduction. Conversely, a standalone five-year drastic reduction of the plastic inflow has a limited impact on the accumulated mass and cannot be seen as a solution to reduce the legacy pollution.

With the performance achieved so far by S03 (around 40% retention efficiency and steering strategy yielding similar results as the HSH scenario), the cleanup objective of removing 80% of the mass of the NPGP baseline be reached after a minimum of 150 system-years with a total cost of 5.3B€. According to the simulations, this goal can be achieved at a much lower cost by increasing retention efficiency from 40% to at least 70% and improving our dispersal modeling so that the quality of our plastic forecast enables an optimized steering strategy. On the one hand, several components of the system can be designed to increase the retention efficiency: the screen can extend deeper than 4 m to prevent plastics from going under the wing, and the floating component can have a higher freeboard to prevent plastics from going above, the length of the lines bearing the tension can also have an impact on the vertical profile of the wing and are points of improvement which are currently being investigated in a series of basin tests. On the other hand, the improvement of the dispersal models rely on: first, a better understanding of plastic transport, which is an ongoing field of research^43^, in particular, the contribution of the three environmental components, sea-surface current, wind and wave- induced drifts for the different plastic types^44,45^; second, a better modeling of those components using assimilated and possibly higher resolution models. Note that the sensitivity of the outcome of these simulations to the circulation model, the inclusion of wind and wave-induced drift in the dispersal model is considered in future work. Finally, a substantial sensing effort using ship-mounted cameras^46^, Unmanned Aerial Vehicles, High Altitude Platforms, and satellites^47,48^ could provide direct surface numerical concentrations, which are also required for model validation and assimilation^49^. In addition, another diagnosis can be performed on near-real-time data from sea-surface elevation and temperature, e.g., high-resolution eddy detection^50^ and Transient Attracting Profiles^51^ to further inform the steering strategy.

With the current performance (S03) and our current knowledge of market rates, the total cost of 150 system-years of operations (15 systems for 10 years) required to remove 80% of the NPGPb baseline scenario is around 5.3B€. This cost could be as low as 1.8B€ if the improvements outlined in the previous paragraph were successful. A minor yet non-negligible portion of this cost could be offset by revenue from recycling NPGP material into durable products, as illustrated in^52^.

Furthermore, an overall environmental balance assessment^31^ indicated that the potential adverse impacts of the cleanup operations (e.g., bycatch and greenhouse gas emissions) are outweighed by the anticipated benefits of removing approximately 80% of meso- and macroplastic debris from the NPGP, including the mitigation of plastic pollution effects on marine ecosystems^53^ and future perturbations to the carbon cycle^54^. The cost of large-scale offshore cleanup operations is often weighed against the cost of cleanup solutions, initiatives, or policies closer to the sources, which are ultimately the best means to tackle future pollution^19^. In the NPGP, the contribution between marine debris originating from fishing or aquaculture activities and originating from land-based sources was estimated at around 80 − 20%^33^. Therefore, coastal/land-based prevention measures will have only a limited impact on the current influx of plastic to this remote region. Conversely, acting on marine sources, especially regarding discarded and derelict fishing gear, shall strengthen and sustain the impact of the offshore cleanup. However, preventing plastic debris from entering the oceans cannot solve the legacy plastic pollution accumulated far offshore in the oceanic garbage patches. Plastic pollution will likely persist at the ocean surfaces for over a century even if inputs cease^55^. Instead of benchmarking the cost of offshore cleanup against of other mitigation measures, it should be evaluated relative to the substantial loss of ecosystem services, which has been estimated to surpass the cost of such initiative by orders of magnitude when accumulated over a century (up to 264 billion USD for the next century for ~ 80,000 tonnes in the GPGP^21^ at 33,000 USD/tonnes/year^22^). In this context, large-scale removal efforts represent not only an environmental intervention but also an economic safeguard against the long-term degradation of marine resources. The benefits of acting now extend far beyond debris removal: they include preserving biodiversity, maintaining the resilience of ocean food webs and avoiding irreversible shifts in biogeochemical cycles. Without proactive measures targeting legacy pollution, the burden of ecological and economic damage will continue to grow, ultimately surpassing the costs of decisive intervention.

## Supplementary Information

Below is the link to the electronic supplementary material.

Supplementary Material 1

## Data Availability

The data used for the NPGP calibration study (system trajectories and encountered wind conditions) along with the NPGP calibration and cleanup algorithms will be made available upon request.
