# Supplementary material for: Modelling the cleanup of the North Pacific Garbage Patch based on 3 years of operational experience
Source: Sci Rep. 2026 Mar 20;16:8050. doi: 10.1038/s41598-026-40859-y (PMC13004986; doi:10.1038/s41598-026-40859-y)
Supplement: Supplementary file 1 — Supplementary Material 1 [file 41598_2026_40859_MOESM1_ESM.docx]

**Supplementary Information for 'Modelling the cleanup of the North Pacific Garbage Patch based on 3 years of operational experience'**

Bruno Sainte-Rose^1*^, Laurent Lebreton^1,2^, Yannick Pham^1^, Arjen Tjallema^1^, Christophe Maes^3^

^1^ The Ocean Cleanup, Rotterdam, The Netherlands

^2^ The Modelling House, Raglan, New Zealand

^3^ Université de Bretagne Occidentale, IRD, Brest, France

* corresponding author (bruno.sainte-rose@theoceancleanup.com)

**Supplementary Methods**

**Supplementary Methods S1: Estimation of extracted plastic mass concentration at the surface**

To derive the averaged extracted plastic mass concentration at the surface $\rho_{n}^{e}$ in (kg/km^2^) for a given

collection period $p$, the total swept area $A_{p}$(km^2^) is obtained by summing the “instantaneous swept areas” over a period going from time $t_{p}^{0}$ to $t_{p}^{f}$ , the starting and ending times of the collection period $p$. The sampling rate of the speed through water is around 1/32 Hz while the GPS locations allowing us to determine the span are measured every 5 minutes; thereby, we take 5 minutes averages of the speed through water ($dt=$300 s).

$$A_{p}=\sum_{{t=t}_{p}^{0}}^{t_{p}^{f}} \Delta\boldsymbol{V}\left( t \right).S\boldsymbol{n}\left( t \right).dt$$

Then, the surface mass concentration of plastics equals $\rho_{p}^{e}=\frac{M_{p}^{e}}{A_{p}}$ where $M_{p}^{e}$ is the mass extracted after a collection period.

**Supplementary Methods S2: Retention efficiency tests**

Field tests were performed during trips 2, 10, 11, 12 and 17. They aimed at evaluating the retention efficiency of the RS and link the extracted plastic mass concentration at the surface to the encountered one. The tests consisted in releasing and recovering 2296 tagged objects in front of the RS span-line. Example of such objects is given in Supplementary Figure S1. The plastics were separated in batches composed of 7 types of plastics: a large ghost net, a medium ghost net, a small ghost net, a crate, one flat hard plastic, an eel trap/bottle, a lid. During those tests the encountered environmental conditions were recorded. Different wind conditions, measured by the ships’ anemometers located above the bridge, were experienced during those tests allowing to evaluate the retention efficiency as a function of wind conditions as shown in Figure 3. The detailed outcome of those tests is given in Supplementary Figure S2.

**Supplementary Methods S3: Plastic dispersal model calibration**

The dispersal model used to simulate the background plastic mass concentration is based on a multi-year experiment with hindcast from 1993 and has been running operationally since August 2021. Lagrangian particles have been released since 1993 from sources along the global coastlines using the scenario [1] and advected using an in-house Lagrangian dispersal model ADVECT [2]. The particles follow the global ocean circulation given by the GLORYSV12 experiment [3]. The model is calibrated (each particle is given a constant weight) such that the average of the "measured" encountered plastic mass concentrations is equal to the modelled one (including the retention efficiency). Supplementary Figure S4 shows a scatter plot between modelled and measured for extracted mass concentrations (panel a) and encountered mass concentrations (panel b).

**Supplementary Methods S4: Wind and wave conditions during collection periods vs conditions in the NPGP**

The measured wind conditions at 10 m altitude experienced by the system during the 103 collection periods are compared statistically to the conditions at point 140°W-30°N in the center of the NPGP for 30 years of reanalysis model data ERA5 [4]. A comparison of the exceedance probabilities for wind magnitude at 10 is given in Supplementary Figure S5 for the 4 system configurations split beween the collection periods that were kept (solid lines) and the discarded ones (dashed lines), and the modelled conditions at the center of the NPGP (circles). The values for the three quartiles (p25, p50 and p75) for the 9 cases are detailed in Supplementary Table S4.

**Supplementary Methods S5: NPGP cleanup modeling**

As introduced in the Methods section, an Eulerian-Lagrangian framework is employed to model the cleanup of the NPGP.

**Background plastic mass concentration model mass balance**

The Lagrangian particles are used, as a first step, to reproduce the baseline evolution of the background plastic in the NPGP over the studied period (2009-2018) and enforce an imposed a.g.r. of the load within the study area (1 or 3%) and, as a second step, track the cleanup levels (between 1 and 0) of the individual particles when the cleanup is applied. The absence of plastic sinks in the global advection model implies that the raw growth in number of particles in the NEP exceeds the required growths (1% and 3%) as shown in Supplementary Figure S6. To reproduce the desired a.g.r., the plastic dispersal data is modified according to the following method, realized for each month of the simulation:

- The number of particles leaving the NEP $N_{out}(t)$ is first processed.
- The number of particles expected in the study region at the end of the month $N_{f}(t)$ is processed from the number at the beginning of the month $N_{i}(t)$ and the required growth rate.
- The number of outside particles allowed to enter the study region is processed: $N_{in}(t) = \left( N_{f}\left( t \right)-N_{i}\left( t \right) \right) + N_{out}(t)$ are then picked randomly.

**RSs motions and cleanup modeling**

The RSs are modeled as $N_{RS}$Lagrangian tracers with the following characteristics: span $S$ (in m), Speed Through Water $\Delta V$ (in m/s), retention efficiency $e_{r}$ (in %) (percentage of the particles entering the system which are extracted), and uptime $e_{ut}$ (in %) (percentage of the time the system is actively collecting). In practice, we take a bundled cleaning efficiency $E=e_{r}.e_{ut}$ (in %). To simulate the cleaning of the NPGP, the following method is applied:

- All the particles ($N_{tot})$ encountering the study region over the 10 years are tracked and affected by a weight coefficient $w_{c}$initiated at 1. At the initial time step, RSs are randomly deployed within the NPGP boundary (zone C in Figure 2).
- The background plastic mass concentration $\rho_{p}(i,j)(t)$ is then processed in the Eulerian framework by summing $w_{c}$ of each particle mapped on the $0.08^{\circ}\times0.08^{\circ}$ grid for the study region (corresponding to a subset of the global GLORYS grid) divided by the grid cell areas (in km^2^). The velocity over ground of the background plastic $\boldsymbol{V}_{P}(i,j)$ is taken as the sea-surface current used to force the plastic dispersal model. The RSs’ displacements are obtained assuming a velocity over ground ($\boldsymbol{V}_{RS})$ equal to the sum of the speed through water and the sea-surface current velocity equal to background plastic velocity: $\boldsymbol{V}_{RS}=$ $\boldsymbol{V}_{P}+$ $\Delta V\boldsymbol{n}$ where $\boldsymbol{n}$ is the unit vector oriented according to the steering strategy. The obtained RS tracks are then mapped into the previous grid. For each RS $p$, the crossing duration is defined for all cells as ${dt}_{RS}(i,j,p)$ such that ${dt}_{RS}\left( i,j,p \right)=0$ if RS $p$ does not cross cell $\left( i,j \right)$ and overall $dt=\sum_{(i,j)} {dt}_{RS}\left( i,j,p \right).$The weight of a particle $q$belonging to a cell $(k,l)$ crossed by RS $p$ is then obtained according to the following equation:

$w_{c}\left( q,t+dt \right) =max(0,w_{c}\left( q,t \right).\left( 1 -E.\Delta V.S.{dt}_{RS}\left( i,j,p \right)./A_{kl} \right))$ (1)

where $A_{kl}$ is the local grid cell surface area.

The effect of the cleaning is then processed: the removed plastic is obtained by summing along the number of particles after considering the effect of all cleanup systems.

**Final evaluation of key parameters**

The evolution of the plastic mass concentration evolution of the area, simulated by applying a fixed weight coefficient $W_{0}$ to all particles obtained by dividing the expected load at the beginning of 2027 by the initial number of particles in the area in the beginning of the simulation period (2009). The application of the above method leads to the following results:

- The encountered plastic mass concentration of RS $p$ is obtained using the weighted averaged $\sum_{\left( i,j \right)} {dt}_{RS}\left( i,j,p \right).W_{0}.\rho_{p}(i,j,t)/dt$.
- The removed mass $M_{r}$ at time $t+dt$ is obtained by taking the difference: $M_{r}\left( t+dt \right)=W_{0}. \sum_{q} w_{c}\left( q,t+dt \right) -w_{c}\left( q,t \right).$
- The remaining mass $M$ at time $t+dt$ is finally processed by direct sum $M\left( t \right)=W_{0}. \sum_{q} w_{c}\left( q,t+dt \right)$.

**Supplementary Methods S5: Steering strategies**

The operational area (which defines the area where one RS can operate) is determined by the white mass concentration iso-line introduced in Figure 2 and corresponds to the equivalent of NPGPb (75% of the mass in NPGPa) of rolling 30-day averages of plastic concentration maps smoothed with a Gaussian filter. Supplementary Figure S7 shows daily (panel a), 30-day rolling average (panel b), and smoothed plastic mass concentrations (>1.5cm) (panel c) along with the NPGPb (equivalent) contour. For each day, the contour's barycenter is computed and referred to as the NPGP center.

The RSs are released at the beginning of the simulation along a radius of 1.5 degrees around the NPGP center. For each iteration, the barrier motion and subsequent cleaning are processed for this period, and the plastic dispersion and concentration maps are processed for the next iteration.

Three strategies are defined for the motion of the RSs:

- Random steering strategy (RND): aims to optimize the spatial spreading of the RSs while remaining near the NPGP center; at each time step (of 12h), 100 random headings between -180° and 180° are generated, and the one that yields the highest separation to the nearest RS is taken. In this scenario, only the contour as defined above is assumed to be known. The RS trajectories are defined to maximize the distance between the RSs to avoid cleaning in the wake of another RS, with the NPGP contour being assimilated as a fake RS.
- Hotspot hunting (HSH): a systematic approach is taken every period (t=3 days). The updated average concentration map is processed for the period, and for each RS, the maximum concentration in reach is selected as a target. Reach is defined as 60% of the period’s traveling distance, based on the propelling speed, and not already affected by another RS in the same period. The system spends the entire period heading towards the target, sweeping back and forth after reaching it the first time. In this scenario, the simulation timestep is set to 12h to accurately represent the S03 radius of turn (180° in 12 h).
- Optimized (OPT): in this scenario a complete knowledge of the concentration is assumed for 7 days forecast. For each period and each RS, the concentration maps along time and barrier locations are fed to a Dynamic Programming path planning algorithm [5], yielding a combination of locations per time step. A speed-over-ground approach is followed to enforce the path despite the action of the sea currents, adapting the span to remain in a constantly swept area. The concentration map given to the other systems is set to zero along the path to avoid multiple affectations.

A sketch of the HSH and OPT scenarios is given in Supplementary Figure S8.

**Supplementary Methods S7: Degradation rates estimations**

To estimate the annual degradation rate of plastics, we applied the following formula [6], which gives the Probability Density Function $m(x,t)$ and size, $s\left( x \right)$, from a starting size $s_{0}$, at time $t$ and fragmentation level $x$:

$m\left( x,t \right)=\frac{\Gamma\left( x+\lambda t \right)}{\Gamma\left( x+1 \right)\Gamma\left( \lambda t \right)}p^{x}{(1-p)}^{\lambda t}$, with $p=0.4$ [7] and $s(x)=s_{0}.{0.5}^{x}$

The relationship between $x$ and $s$ will then read as $x(s,s_{0})=-log(\frac{s}{s_{0}})/log(2)$. To simulate both slow and fast degrading plastics, values of $\lambda$ ranging from 0.05 to 0.0005 /year were considered. To simulate the annual degradation rate of plastics (>1.5 cm) to (0.5-1.5 cm) and from (0.5-1.5 cm) to (<0.5 cm), the (>1.5 cm) size class was split into 4 intermediate size classes: (1.5–5 cm), (5-15 cm), (15-50 cm), (>50 cm). The initial mass distribution of plastics within the (>1.5 cm) size class is the following: 14%, 17%, 32%, 37% (designated by $\alpha_{i=1,4}$) as per a recent study [7].

Supplementary Figure S9 shows the distribution of $m$ as a function of $s$ for $t=1$year. From these distributions, one can derive the annual degradation rate $r$ as the following: for the degradation rate from (>1.5 cm) to(0.5-1.5 cm): $r=\sum_{i=1}^{4} \alpha_{i}\int_{s=0.5cm}^{s=1.5cm} m\left( x\left( s,s_{0}^{i} \right),t= 1 year \right).dx$ where $s_{0}^{1}$=1 cm, $s_{0}^{1}$=3.25 cm, $s_{0}^{2}$=10 cm, $s_{0}^{3}$= 32.5 cm, $s_{0}^{4}$ = 75 cm correspond to the center of the 4 size classes bins; for the degradation rate from (0.5-1.5 cm) to (<0.5 cm): $r=\int_{s=0 cm}^{s=0.5cm} m\left( x\left( s,s_{0} \right),t= 1 year \right).dx$ where $s_{0}$=1 cm.

Finally the computed annual degradation rates from (>1.5 cm) to (0.5-1.5 cm) are 1.2 resp. 0.4% for $\lambda$ =0.05/y resp. 0.0005/y; from (0.5-1.5 cm) are 6 resp. 2% for $\lambda$ =0.05/y resp. 0.0005/y.

**Supplementary Methods S8: Cost comparison between modelled scenarios and experimented system configurations**

Supplementary Figure S13 shows a comparison of the full cleanup cost (OPEX + CAPEX) for S002-S03 and S0X systems (with X>3) which correspond to the systems used in the future with the 3 different steering strategies. The performances considered for S002-S03 correspond to the ones experienced during the 2021-2024 operations in terms of span, speed through water, extracted plastic mass and uptime (see Supplementary Tables S1, S2 and S5). The modelled scenarios of future systems include an improved uptime (80%), larger span (1800 m) and increased retention efficiency (70%).

**Supplementary Figures**


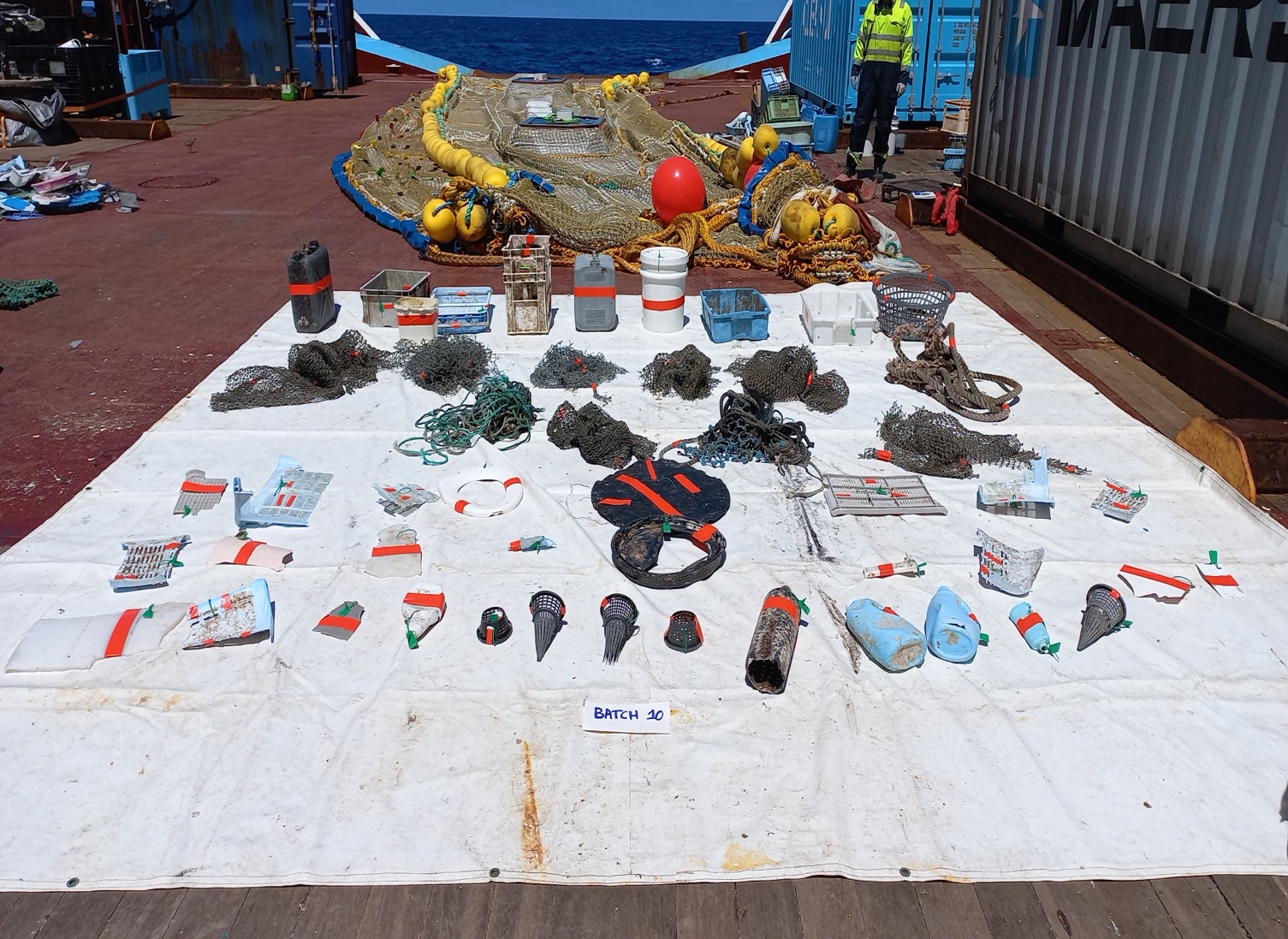


Supplementary Figure S1: Picture of a batch of tagged plastics used for a retention efficiency test


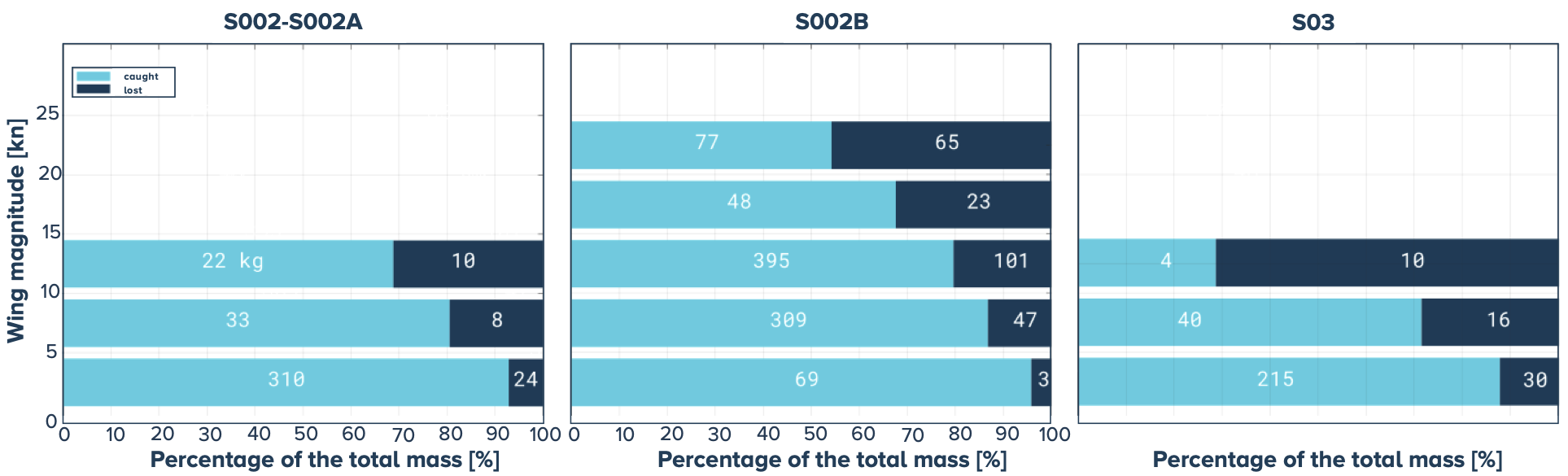


Supplementary Figure S2: Outcome of the retention efficiency tests binned per wind speed (in knots) for the three system versions, mass of caught/lost objects inside the colorbars (in kg).


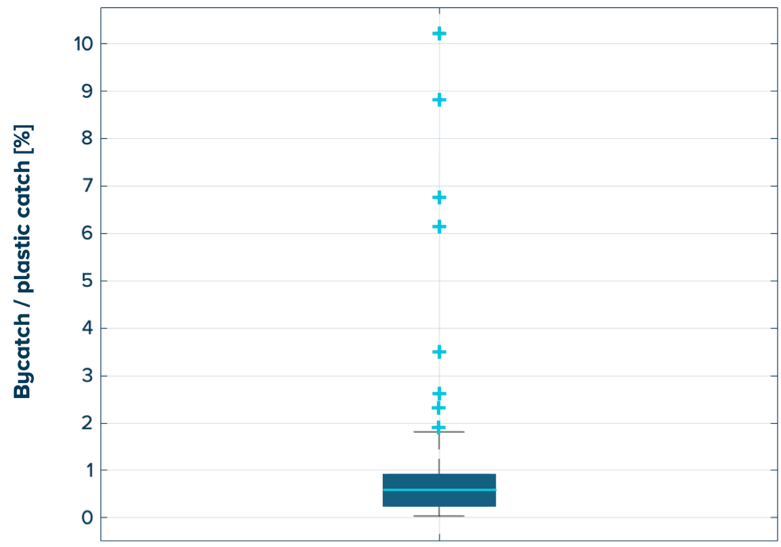


Supplementary Figure S3: Boxplot of the ratio between the weight of the bycatch and the plastic in percentage.


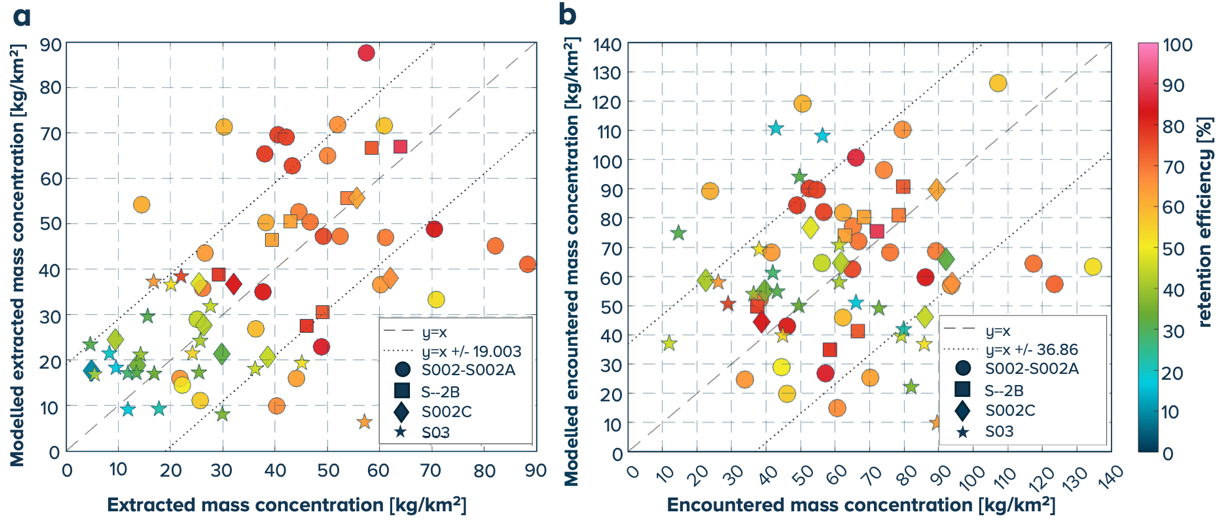


Supplementary Figure S4: Model calibration results: (**a**) scatter plot of extracted plastic mass concentrations: measured vs. model extracted plastic mass colored by modeled retention efficiency for the 72 collection periods; (**b**) scatter plot of encountered mass plastic mass concentrations: measured vs. modelled, colored by modeled retention efficiency. The meaning of the symbols is given in the legend.


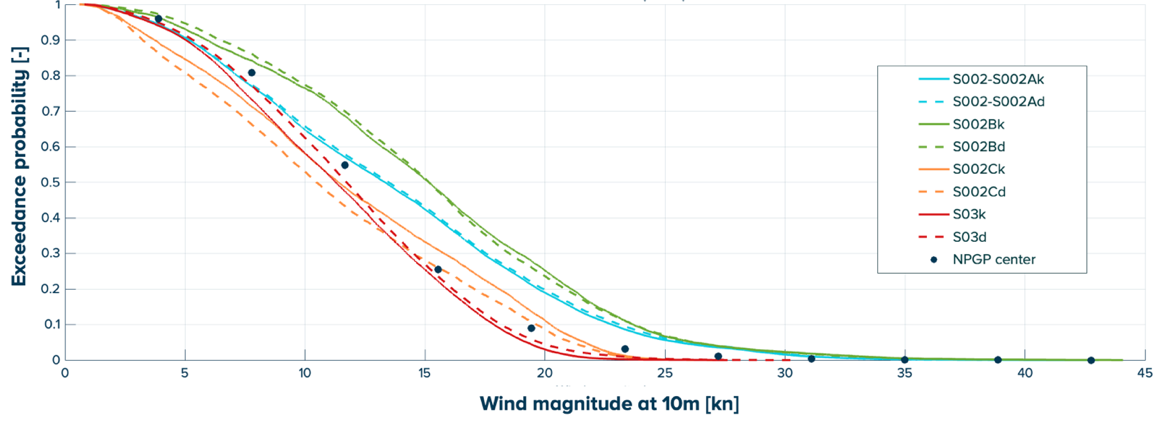


*Supplementary Figure S5: Exceedance probability for wind magnitude at 10m, solid lines: measurements from the kept collection periods (suffix k), dashed lines: measurements from the discarded collection periods (suffix d), plain circles: 30 years reanalysis model in the NPGP center (ERA5).*


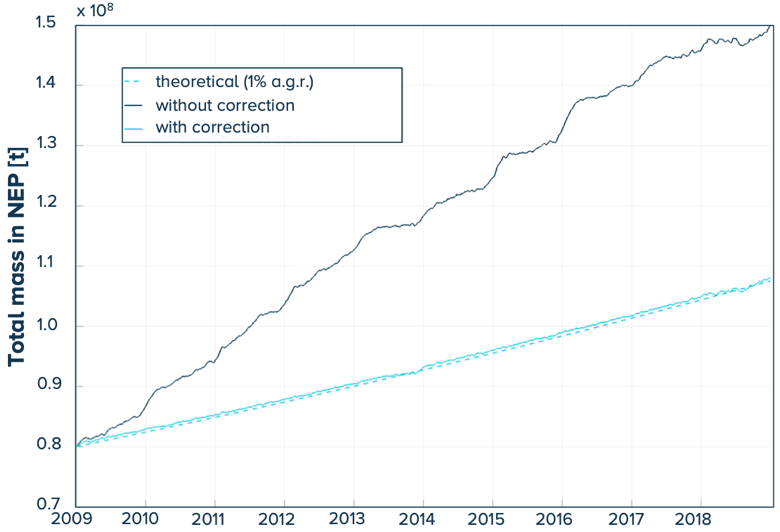


Supplementary Figure S6: Evolution of the number of particles from the global dispersal model solid lines (with and without correction in the NEP) vs. the required number of particles dashed line.


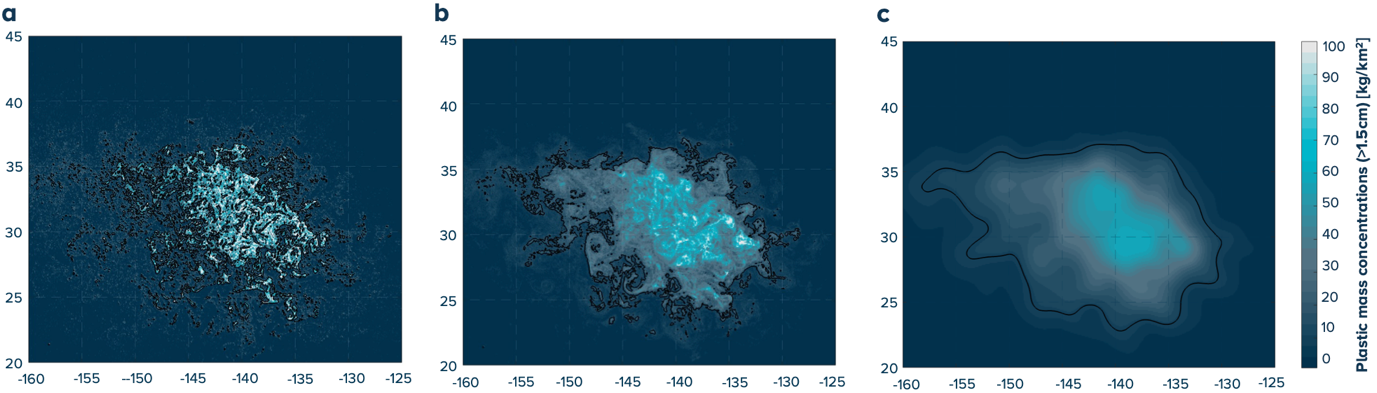


*Supplementary Figure S7: Examples of plastic concentration maps for different averaging techniques: (****a****) plastic mass concentration (in kg/km^2^) for a daily particle projection, (****b****) for 30-day rolling averages, (****c****) Gaussian smoothed version of (****b****); black lines: NPGPb contour (75% of the whole mass in NPGPa grid).*


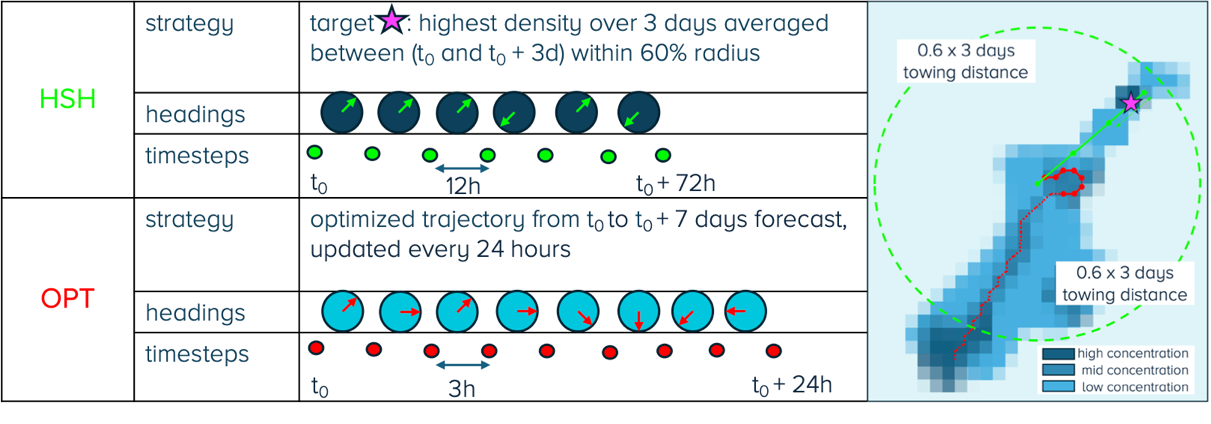


*Supplementary Figure S8: schematic description of the HSH and OPT steering strategies.*


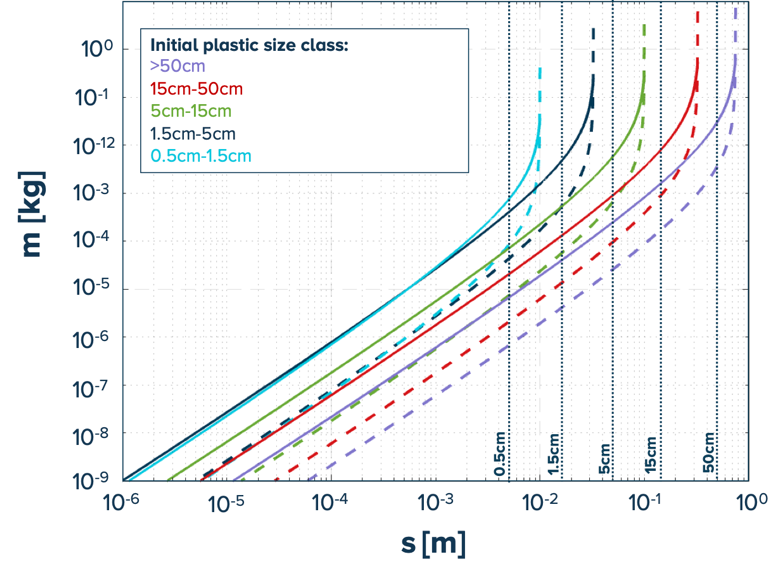


*Supplementary Figure S9: Evolution of the mass PDF after 1 year of degradation m(s, t = 1 year), for the 5 size classes considered, with colors in the legend. Solid lines: λ = 0.05 /y and dashed lines λ = 0.0005 /y.*


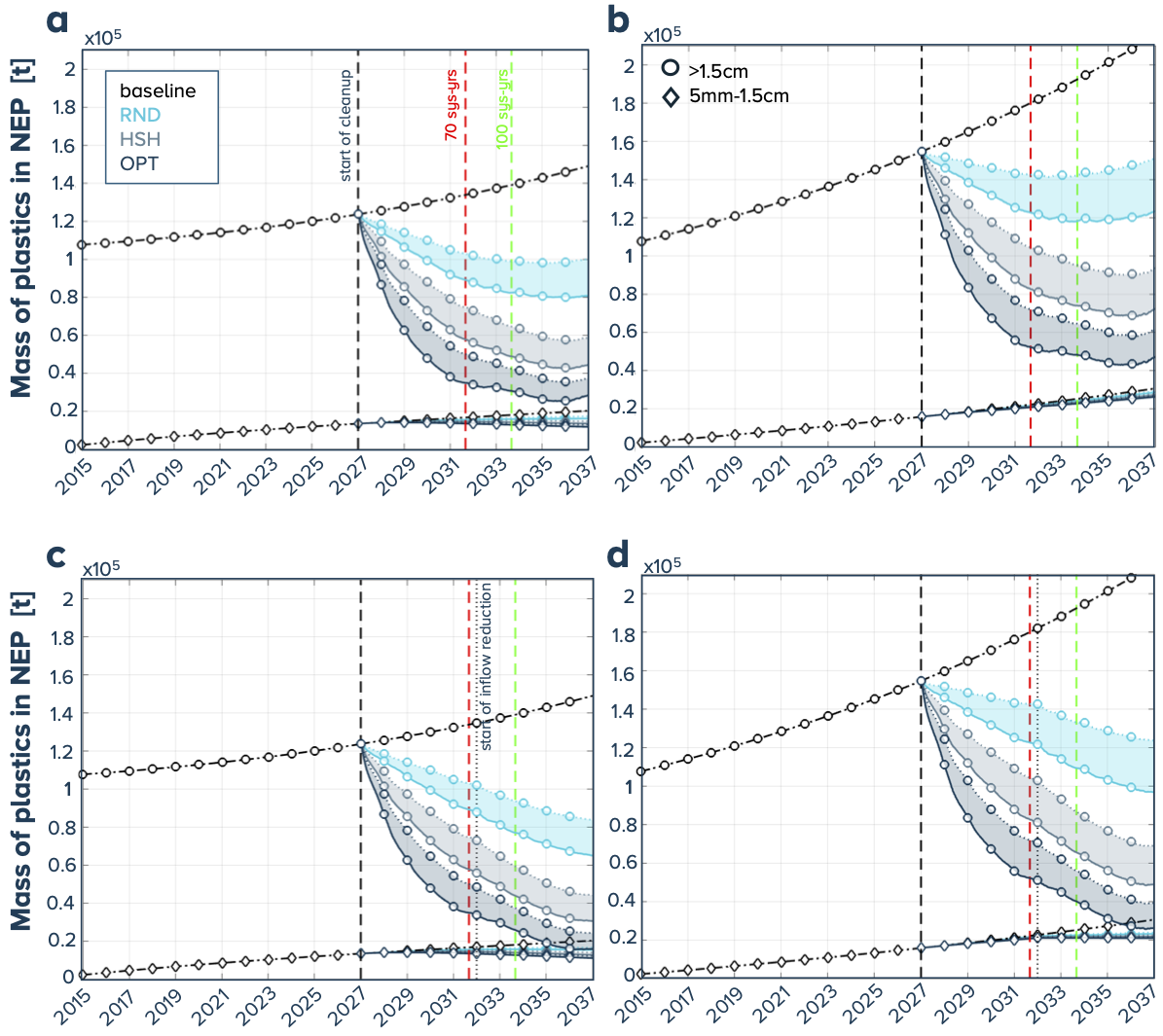


*Supplementary Figure S10: Evolution of the mass inside the NEP region (in tonnes) for the different steering strategies: RND for random steering, HSH for hotspot-hunting and OPT for optimized (corresponding colors given in panel a) and the two size classes (corresponding symbols given in panel b) for 15 cleanup systems starting in 2015: (solid lines: retention efficiency 0.7, dotted lines retention efficiency 0.4, (****a****,* ***c****) resp. (****b****,* ***d****) correspond to 1% resp. 3% a.g.r. of (>1.5cm) plastics and from top to bottom row: offshore cleanup only (****a****,* ***b****), the combination of inflow reduction and offshore cleanup (****c****,* ***d****).*


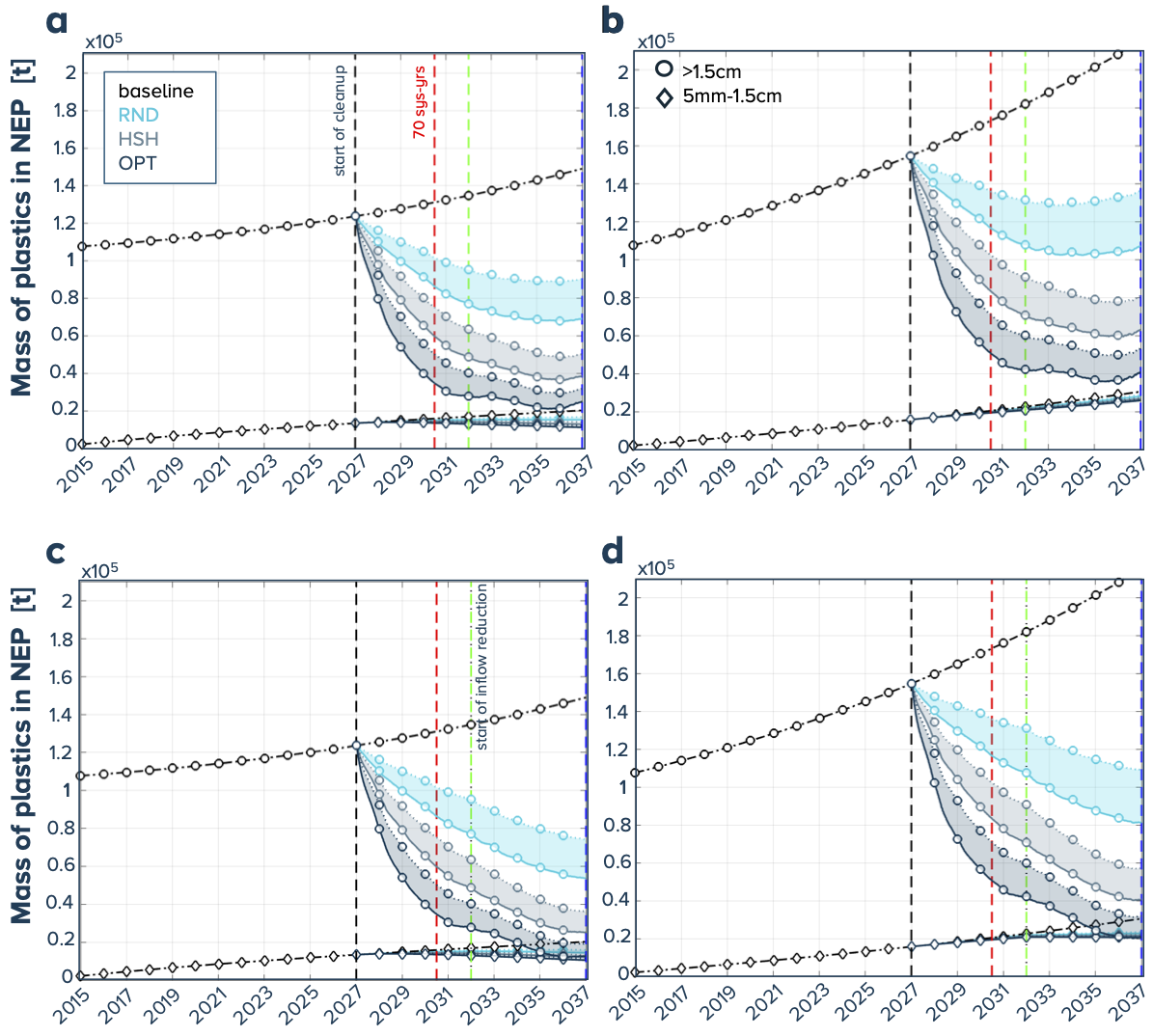


*Supplementary Figure S11: Evolution of the mass inside the NEP region (in tonnes) for the different steering strategies: RND for random steering, HSH for hotspot-hunting and OPT for optimized (corresponding colors given in panel a) and the two size classes (corresponding symbols given in panel b) for 20 cleanup systems starting in 2015 (solid lines: retention efficiency 0.7, dotted lines retention efficiency 0.4). (****a****,* ***c****) resp. (****b****,* ***d****) correspond to 1% resp. 3% a.g.r. of (>1.5cm) plastics and from top to bottom row: offshore cleanup only (****a****,* ***b****), the combination of inflow reduction and offshore cleanup (****c****,* ***d****).*

*
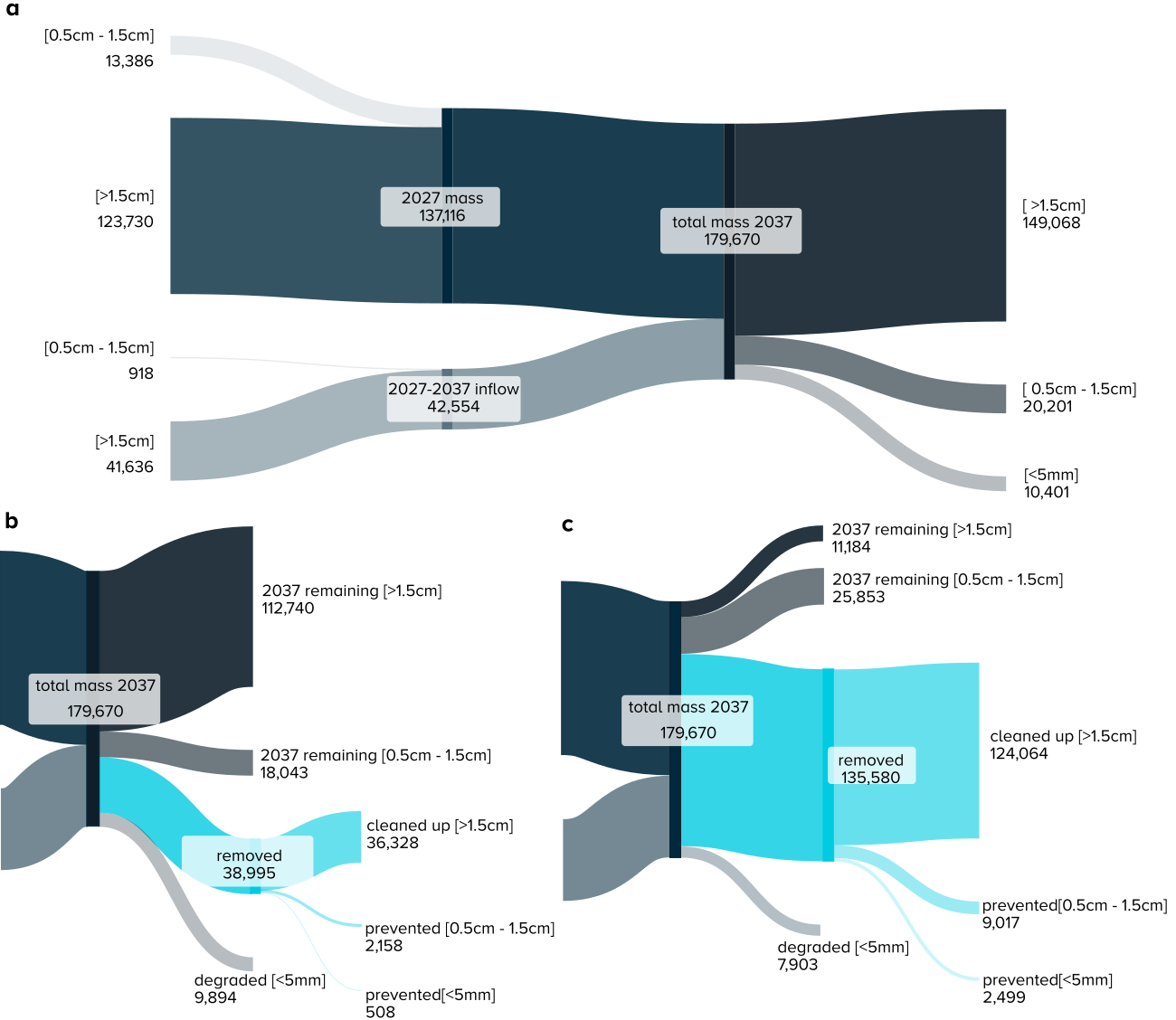
*

*Supplementary Figure S12: Sankey diagrams showing the evolution of the total mass of the different size classes (in t) for (****a****) the baseline scenario for 1% a.g.r., (****b****) the worst case (random steering strategy and 40% retention efficiency) and (****c****) the best case (optimized steering strategy and 70% retention efficiency) for 1% a.g.r.*

**
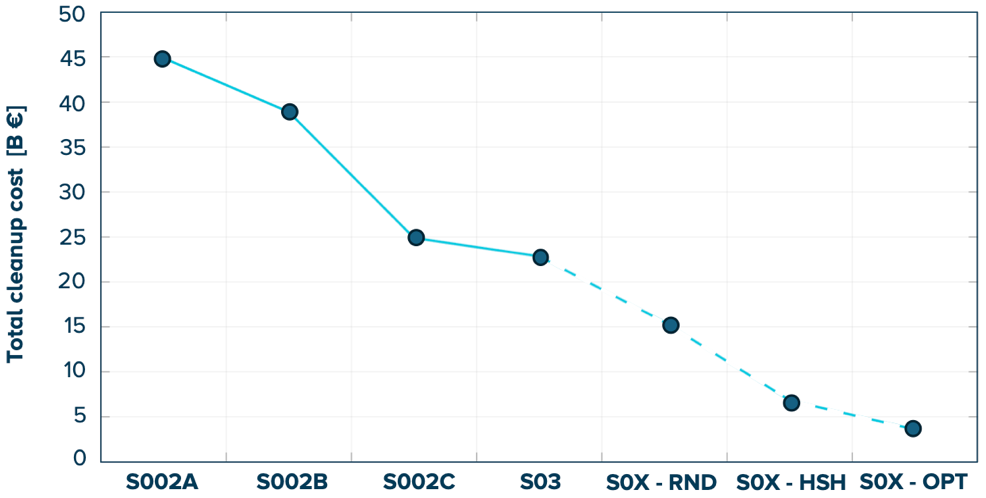
**

*Supplementary Figure S13: Evolution of the total projected cleanup depending on system versions based on experienced costs of S002A, S002B, S002C and S03. S0X-RND, S0X-HSH and S0X-OPT are based on the current study results (improved uptime, improved retention efficiency for the three steering strategy scenarios).*

**Supplementary Tables**

*Supplementary Table S1: Main characteristics of the five system version.*

| **System version** | **Nominal span** **[m]** | **Wing depth** **[m]** | **Mesh size of the net**  **[cm]** | **RZ capacity** **[t]** | **Collection periods** |
| --- | --- | --- | --- | --- | --- |
| S002 | 500 | 3 | 1 | 15 | 1-32 |
| S002A | 500 | 3 | 1 | 25 | 33-44 |
| S002B | 550 | 4 | 1.6 | 25 | 45-54 |
| S002C | 1100 | 4 | 1.6 | 25 | 55-67 |
| S03 | 1450 | 4 | 1.6 | 25 | 68-103 |

*Supplementary Table S2: Information on the collection periods considered in the dispersal model calibration.*

| **#** | **System version** | **Time start** | **Time end** | **Swept area** **[km2]** | **Rigids (>5cm)** **[kg]** | **Fibrous (>5cm)**  **[kg]** | **Waste (>1.5cm)** **[kg]** | **Total catch** **[kg]** | **Bycatch**  **[kg]** |
| --- | --- | --- | --- | --- | --- | --- | --- | --- | --- |
| 1 | S002 | 09/08/2021 21:55 | 10/08/2021 13:50 | 1.5 | 35 | 50 | 23 | 108 | 7 |
| 2 | S002 | 19/08/2021 07:00 | 20/08/2021 15:45 | 11.3 | 30 | 78 | 17 | 125 | 8 |
| 5 | S002 | 22/08/2021 18:10 | 24/08/2021 17:25 | 35.7 | 1273 | 1273 | 21 | 2567 | 5 |
| 6 | S002 | 18/09/2021 01:50 | 22/09/2021 12:00 | 88.1 | 1304 | 2639 | 27 | 3970 | 5 |
| 7 | S002 | 23/09/2021 22:40 | 25/09/2021 14:00 | 32.1 | 702 | 809 | 178 | 1689 | 3 |
| 8 | S002 | 25/09/2021 19:30 | 08/10/2021 18:25 | 249.6 | 3287 | 5265 | 374 | 8926 | 20 |
| 9 | S002 | 10/10/2021 02:40 | 13/10/2021 21:00 | 72.3 | 1554 | 2320 | 829 | 4703 | 17 |
| 11 | S002 | 08/11/2021 18:15 | 18/11/2021 17:30 | 124.7 | (-) | (-) | 3345 | 3345 | 8 |
| 12 | S002 | 17/12/2021 02:15 | 17/12/2021 19:30 | 18.4 | 212 | 210 | 0 | 422 | 2 |
| 13 | S002 | 18/12/2021 00:30 | 27/12/2021 01:00 | 147.2 | 667 | 3590 | 1 | 4258 | 3 |
| 15 | S002 | 27/12/2021 23:10 | 01/01/2022 14:50 | 35.2 | 711 | 1796 | 2 | 2509 | 88 |
| 17 | S002 | 22/01/2022 23:50 | 27/01/2022 17:45 | 73.7 | 934 | 2399 | 146 | 3479 | 29 |
| 18 | S002 | 29/01/2022 23:15 | 05/02/2022 16:50 | 126.9 | 1614 | 2489 | 125 | 4228 | 40 |
| 20 | S002 | 07/02/2022 03:25 | 14/02/2022 15:00 | 140.6 | 1757 | 4993 | 322 | 7072 | 24 |
| 21 | S002 | 09/03/2022 22:30 | 17/03/2022 16:05 | 151.4 | 388 | 1068 | 42 | 1498 | 14 |
| 25 | S002 | 17/03/2022 23:10 | 23/03/2022 17:30 | 110.4 | 599 | 1129 | 33 | 1761 | 12 |
| 26 | S002 | 30/04/2022 17:15 | 03/05/2022 17:00 | 84.2 | 1161 | 2656 | 300 | 4117 | 95 |
| 27 | S002 | 04/05/2022 00:00 | 04/05/2022 19:30 | 20.5 | 300 | 464 | 79 | 843 | 8 |
| 28 | S002 | 05/05/2022 00:00 | 08/05/2022 19:50 | 25.0 | 383 | 904 | 71 | 1358 | 8 |
| 29 | S002 | 26/05/2022 02:00 | 30/05/2022 14:55 | 130.8 | 1452 | 2927 | 199 | 4578 | 14 |
| 30 | S002 | 31/05/2022 00:20 | 05/06/2022 18:50 | 143.2 | 2203 | 4235 | 290 | 6728 | 27 |
| 31 | S002 | 06/06/2022 16:00 | 08/06/2022 14:30 | 43.4 | 542 | 914 | 133 | 1589 | 14 |
| 32 | S002 | 08/06/2022 19:05 | 13/06/2022 15:20 | 129.5 | 648 | 1206 | 29 | 1883 | 6 |
| 33 | S002A | 13/06/2022 19:40 | 18/06/2022 15:30 | 103.2 | 736 | 875 | 35 | 1646 | 7 |
| 34 | S002A | 06/07/2022 01:30 | 10/07/2022 19:00 | 111.8 | 3099 | 4513 | 175 | 7787 | 17 |
| 35 | S002A | 11/07/2022 15:00 | 16/07/2022 16:00 | 117.6 | 2768 | 4670 | 248 | 7686 | 21 |
| 36 | S002A | 17/07/2022 00:00 | 21/07/2022 14:25 | 100.8 | 2405 | 3684 | 230 | 6319 | 13 |
| 37 | S002A | 21/07/2022 21:30 | 27/07/2022 14:25 | 130.6 | 2018 | 3504 | 163 | 5685 | 11 |
| 40 | S002A | 27/07/2022 21:10 | 30/07/2022 17:20 | 50.8 | 1234 | 2148 | 125 | 3507 | 9 |
| 41 | S002A | 22/08/2022 15:00 | 31/08/2022 19:20 | 140.1 | 2176 | 3986 | 457 | 6619 | 12 |
| 42 | S002A | 01/09/2022 04:00 | 05/09/2022 16:20 | 78.5 | 2114 | 4244 | 510 | 6868 | 6 |
| 44 | S002A | 06/09/2022 03:20 | 12/09/2022 15:45 | 170.3 | 3134 | 4854 | 588 | 8576 | 3 |
| 46 | S002B | 04/10/2022 22:00 | 11/10/2022 18:10 | 181.3 | 3644 | 6118 | 324 | 10086 | 11 |
| 47 | S002B | 19/10/2022 00:00 | 24/10/2022 13:00 | 161.3 | 3848 | 6656 | 251 | 10755 | 28 |
| 48 | S002B | 12/11/2022 22:15 | 17/11/2022 15:45 | 163.8 | 1810 | 2775 | 411 | 4996 | 15 |
| 49 | S002B | 18/11/2022 01:00 | 23/11/2022 01:50 | 79.9 | 1750 | 3213 | 385 | 5348 | 41 |
| 50 | S002B | 23/11/2022 20:45 | 28/11/2022 16:20 | 122.8 | 2307 | 3580 | 309 | 6196 | 47 |
| 52 | S002B | 29/11/2022 00:00 | 05/12/2022 00:05 | 164.7 | 2708 | 4430 | 512 | 7650 | 56 |
| 53 | S002B | 01/04/2023 00:55 | 05/04/2023 14:55 | 146.9 | 2510 | 2397 | 800 | 5707 | 103 |
| 55 | S002B | 05/04/2023 21:40 | 08/04/2023 17:00 | 78.0 | 990 | 945 | 210 | 2145 | 7 |
| 56 | S002C | 29/04/2023 21:10 | 03/05/2023 15:50 | 168.6 | 1862 | 2358 | 447 | 4667 | 37 |
| 57 | S002C | 03/05/2023 23:50 | 08/05/2023 15:35 | 184.6 | 2140 | 3783 | 868 | 6791 | 28 |
| 60 | S002C | 09/05/2023 23:00 | 12/05/2023 15:25 | 119.6 | 1484 | 2495 | 571 | 4550 | 42 |
| 61 | S002C | 08/06/2023 14:30 | 15/06/2023 14:35 | 383.5 | 2285 | 5000 | 884 | 8169 | 61 |
| 62 | S002C | 16/06/2023 01:30 | 21/06/2023 21:50 | 349.9 | 2475 | 3672 | 39 | 6186 | 9 |
| 63 | S002C | 22/06/2023 03:30 | 27/06/2023 14:35 | 121.9 | 1623 | 4163 | 1002 | 6788 | 10 |
| 64 | S002C | 28/06/2023 01:00 | 28/06/2023 18:15 | 30.7 | 237 | 348 | 51 | 636 | 9 |
| 65 | S002C | 28/06/2023 21:00 | 01/07/2023 14:30 | 118.9 | 644 | 1471 | 132 | 2247 | 8 |
| 66 | S002C | 22/07/2023 15:05 | 25/07/2023 19:40 | 150.0 | 1528 | 3213 | 760 | 5501 | 98 |
| 68 | S002C | 26/07/2023 02:15 | 28/07/2023 16:40 | 161.0 | 1575 | 2118 | 247 | 3940 | 34 |
| 69 | S03 | 02/08/2023 22:10 | 07/08/2023 16:55 | 309.9 | 3063 | 7077 | 1213 | 11353 | 31 |
| 70 | S03 | 08/08/2023 02:55 | 10/08/2023 15:50 | 167.0 | 1847 | 3116 | 360 | 5323 | 44 |
| 71 | S03 | 29/08/2023 20:20 | 04/09/2023 14:50 | 295.8 | 2274 | 3653 | 992 | 6919 | 14 |
| 72 | S03 | 05/09/2023 01:00 | 12/09/2023 17:15 | 492.5 | 5383 | 10500 | 2477 | 18360 | 12 |
| 73 | S03 | 14/09/2023 03:30 | 18/09/2023 14:45 | 216.6 | 1526 | 5843 | 961 | 8330 | 151 |
| 75 | S03 | 18/09/2023 20:45 | 22/09/2023 15:10 | 303.5 | 2645 | 3364 | 513 | 6522 | 54 |
| 76 | S03 | 14/10/2023 15:20 | 17/10/2023 22:35 | 109.7 | 791 | 941 | 115 | 1847 | 21 |
| 78 | S03 | 18/10/2023 17:40 | 25/10/2023 14:40 | 559.8 | 4565 | 6903 | 406 | 11874 | 36 |
| 79 | S03 | 27/10/2023 15:35 | 29/10/2023 15:20 | 106.5 | 442 | 467 | 64 | 973 | 26 |
| 82 | S03 | 29/10/2023 17:00 | 01/11/2023 16:00 | 172.5 | 1351 | 1626 | 184 | 3161 | 24 |
| 85 | S03 | 16/03/2024 01:30 | 18/03/2024 19:10 | 155.2 | 1434 | 2820 | 351 | 4605 | 28 |
| 86 | S03 | 24/04/2024 20:20 | 26/04/2024 19:30 | 37.4 | 113 | 95 | 29 | 237 | 24 |
| 87 | S03 | 26/04/2024 22:35 | 03/05/2024 12:30 | 430.3 | 2125 | 4410 | 880 | 7415 | 45 |
| 88 | S03 | 05/05/2024 01:20 | 10/05/2024 19:50 | 464.9 | 2928 | 4362 | 706 | 7996 | 55 |
| 89 | S03 | 11/05/2024 20:45 | 16/05/2024 14:45 | 338.8 | 2163 | 5462 | 606 | 8231 | 48 |
| 91 | S03 | 17/05/2024 01:00 | 20/05/2024 14:35 | 180.2 | 869 | 1882 | 325 | 3076 | 28 |
| 93 | S03 | 13/06/2024 18:05 | 26/06/2024 15:00 | 948.4 | 5542 | 9046 | 1622 | 16210 | 312 |
| 94 | S03 | 05/07/2024 00:05 | 12/07/2024 14:45 | 481.5 | 2326 | 7045 | 965 | 10336 | 155 |
| 99 | S03 | 12/07/2024 23:45 | 18/07/2024 00:00 | 231.0 | 1158 | 2743 | 286 | 4187 | 41 |
| 100 | S03 | 18/09/2024 17:30 | 22/09/2024 15:00 | 141.3 | 350 | 901 | 64 | 1315 | 6 |
| 102 | S03 | 22/09/2024 21:10 | 30/09/2024 21:20 | 533.3 | 2833 | 5753 | 1728 | 10314 | 39 |
| 103 | S03 | 08/10/2024 03:00 | 21/10/2024 18:00 | 831.9 | 1528 | 4548 | 625 | 6701 | 9 |

*Supplementary Table S3: Cause of failure for the 31 collection periods that were not used in this study and were discarded in the dispersion model calibration*

| **#** | **System version** | **Extraction date (UTC)** | **Total catch [kg]** | **Causes of failure** |
| --- | --- | --- | --- | --- |
| 3 | S002 | 27/08/2021 18:48 | 3180 | DVL-STW data unreliable |
| 4 | S002 | 31/08/2021 15:45 | 1398 | Unavailable span data for ~20% of the collection period |
| 10 | S002 | 23/11/2021 17:50 | 1483 | Broken zipper near the RZ |
| 14 | S002 | 22/01/2022 19:30 | 0 | Emergency release |
| 16 | S002 | 29/01/2022 21:15 | 0 | Emergency release |
| 19 | S002 | 08/03/2022 15:45 | 1089 | Faulty data due to emergency release (open system) |
| 22 | S002 | 29/03/2022 15:40 | 0 | Emergency release |
| 23 | S002 | 22/04/2022 21:21 | 631 | Single vessel tow through storm |
| 24 | S002 | 30/04/2022 01:33 | 0 | Emergency release |
| 38 | S002A | 19/08/2022 15:40 | 133 | Wing fully twisted |
| 39 | S002A | 22/08/2022 14:35 | 3082 | Open connection starboard wing near RZ |
| 43 | S002B | 04/10/2022 14:00 | 5277 | Vertical wing shape issue |
| 45 | S002B | 17/10/2022 17:11 | 2496 | System choked |
| 51 | S002B | 31/03/2023 15:07 | 4661 | Unreliable swept area: wrong trackers deployed |
| 54 | S002C | 29/04/2023 16:57 | 0 | Emergency release |
| 58 | S002C | 19/05/2023 15:18 | 700 | Accidental emergency release |
| 59 | S002C | 20/05/2023 15:20 | 2928 | Catch coming from previous emergency release |
| 67 | S002C | 01/08/2023 16:07 | 5699 | Unreliable swept area: wrong trackers deployed |
| 74 | S03 | 27/09/2023 20:42 | 5717 | Cod-end knot wing module 3 portside found open |
| 77 | S03 | 27/10/2023 15:03 | 0 | Emergency release |
| 80 | S03 | 07/11/2023 22:37 | 0 | Emergency release |
| 81 | S03 | 14/03/2024 17:38 | 7029 | Major leaks observed through holes |
| 83 | S03 | 25/03/2024 18:41 | 9144 | System choked + failed wing-to-wing connection |
| 90 | S03 | 29/05/2024 16:18 | 6512 | Large amount of material lost during extraction |
| 84 | S03 | 08/04/2024 15:35 | 6433 | Failed wing-to-wing connection, wing damages |
| 92 | S03 | 04/07/2024 14:48 | 7194 | Hole on wing module 3 starboard |
| 95 | S03 | 09/08/2024 15:05 | 7861 | Hole on wing module 10 |
| 96 | S03 | 16/08/2024 19:23 | 5490 | System choked |
| 97 | S03 | 24/08/2024 14:55 | 5393 | Retention Hatch System inflated for 36 hours |
| 98 | S03 | 31/08/2024 16:10 | 3657 | Consequent damages on first 8 wing modules |
| 101 | S03 | 07/10/2024 19:00 | 4657 | Hole in wing module 4 |

*Supplementary Table S4: Measured wind magnitude quartiles (in knots) for the 4 systems versions for the kept (k) and discarded collection periods and the 30-year reanalysis model at the NPGP center.*

| **System version** | **Wind magnitude quartiles [kn]** | | |
| --- | --- | --- | --- |
|  | **p25** | **p50** | **p75** |
| S002-S002Ak | 8.2 | 13.3 | 18.6 |
| S002-S002Ad | 8.3 | 13.5 | 18.7 |
| S002Bk | 10.4 | 15.2 | 20.0 |
| S002Bd | 10.6 | 15.2 | 19.7 |
| S002Ck | 7.1 | 11.4 | 16.9 |
| S002Cd | 6.2 | 10.5 | 15.8 |
| S03k | 7.5 | 11.3 | 15.1 |
| S03d | 8.1 | 11.7 | 15.3 |
| NPGP center | 8.4 | 12.3 | 15.6 |

*Supplementary Table S5: Breakdown of hours spent in the field (from initial deployment until the end of final recovery) and hours spent collecting plastics to derive uptime per trip number.*

| **Trip** | **Start date (UTC)** | **End date (UTC)** | **In-field [hrs]** | **Collecting [hrs]** | **Uptime [%]** |
| --- | --- | --- | --- | --- | --- |
| 1 | 28/07/2021 20:00 | 08/09/2021 20:00 | 638 | 134 | 21 |
| 2 | 08/09/2021 20:00 | 20/10/2021 20:00 | 676 | 518 | 77 |
| 3 | 20/10/2021 20:00 | 01/12/2021 20:00 | 466 | 193 | 41 |
| 4 | 01/12/2021 20:00 | 12/01/2022 20:00 | 540 | 274 | 51 |
| 5 | 12/01/2022 20:00 | 23/02/2022 20:00 | 680 | 483 | 71 |
| 6 | 23/02/2022 20:00 | 06/04/2022 20:00 | 702 | 428 | 61 |
| 7 | 06/04/2022 20:00 | 18/05/2022 20:00 | 635 | 272 | 43 |
| 8 | 18/05/2022 20:00 | 28/06/2022 20:00 | 604 | 473 | 78 |
| 9 | 28/06/2022 20:00 | 10/08/2022 20:00 | 643 | 532 | 83 |
| 10 | 10/08/2022 20:00 | 20/09/2022 20:00 | 636 | 418 | 66 |
| 11 | 20/09/2022 20:00 | 02/11/2022 20:00 | 658 | 444 | 68 |
| 12 | 02/11/2022 20:00 | 14/12/2022 20:00 | 633 | 404 | 64 |
| 13 | 08/03/2023 20:00 | 19/04/2023 20:00 | 605 | 323 | 53 |
| 14 | 19/04/2023 20:00 | 31/05/2023 20:00 | 632 | 479 | 76 |
| 15 | 31/05/2023 20:00 | 12/07/2023 20:00 | 635 | 476 | 75 |
| 16 | 12/07/2023 20:00 | 23/08/2023 20:00 | 634 | 379 | 60 |
| 17 | 23/08/2023 20:00 | 04/10/2023 20:00 | 732 | 548 | 75 |
| 18 | 04/10/2023 20:00 | 15/11/2023 20:00 | 631 | 494 | 78 |
| 19 | 28/02/2024 20:00 | 17/04/2024 20:00 | 779 | 512 | 66 |
| 20 | 17/04/2024 20:00 | 05/06/2024 20:00 | 874 | 621 | 71 |
| 21 | 05/06/2024 20:00 | 24/07/2024 20:00 | 870 | 699 | 80 |
| 22 | 24/07/2024 20:00 | 11/09/2024 20:00 | 917 | 638 | 70 |
| 23 | 11/09/2024 20:00 | 30/10/2024 20:00 | 827 | 728 | 88 |

*Supplementary Table S6: Parameters used to determine the cost of the cleanup of the NPGP.*

| **Cost items** | **Unit** | **Value** |
| --- | --- | --- |
| ***CAPEX*** |  | |
| Vessel purchase (inc. outfitting and mobilization) | (m€/vessel) | 14.3 |
| Complete retention system (incl. Electrical and Instrumentation) | (m€/system) | 4.60 |
| Additional retention systems for Inspection Repairs and Maintenance | (%) | 25% |
| CAPEX hotspot detection - whole fleet | (m€/fleet) | 10 |
| CAPEX hotspot detection - per system | (m€/system) | 1.50 |
| ***OPEX*** |  | |
| OPEX hotspot detection - whole fleet | (m€/fleet) | 15 |
| OPEX hotspot detection - per vessel | (m€/system) | 1.00 |
| Yearly cost of spares and replacement parts (rel. to CAPEX) | (%) | 10% |
| Project crew (marine, offshore manager, cooks, etc.) | (€/vessel/day) | 1,750 |
| Other crew (Offshore Representative, Protected Species Observer) + small costs | (€/vessel/day) | 2,000 |
| Fuel price | (€/mt) | 1,050 |
| Cost CO2 offsetting | (€/mt) | 15.00 |
| Cost of port call | (€/vessel/port call) | 110,000 |
| Waste processing / recycling | (€/kg) | 1.74 |
| Vessel Management Service | (€/vessel/day) | 11,318 |
| ***OTHER*** |  | |
| Overhead (including FTEs) | (m€/year) | 27.8 |
| Yearly inflation | (%) | 4% |

*Supplementary Table S7: Outcome of the cleanup after 10 years for the 36 scenarios. Nomenclature for scenario naming: AWNXXYYYZZ, where W is the a.g.r., XX is the number of systems (10/15/20), YYY the steering strategy, and ZZ the retention efficiency (40/70).*

| Case | **Total mass in NEP w/ cleanup** | | | **Total mass in NEP baseline** | | | **Net inflow** | |
| --- | --- | --- | --- | --- | --- | --- | --- | --- |
|  | >1.5cm [t] | 0.5-1.5cm [t] | <0.5cm [t] | >1.5cm [t] | 0.5-1.5cm [t] | <0.5cm [t] | >1.5cm [t] | 0.5-1.5cm [t] |
| A1N10RND40 | *112740* | *18043* | *9893* | 149068 | 20201 | 10402 | 41636 | 918 |
| A1N10RND70 | *96052* | *17049* | *9662* |  |  |  |  |  |
| A1N15RND40 | *100174* | *17306* | *9725* |  |  |  |  |  |
| A1N15RND70 | *80750* | *16073* | *9416* |  |  |  |  |  |
| A1N20RND40 | *90097* | *16685* | *9570* |  |  |  |  |  |
| A1N20RND70 | *68971* | *15188* | *9173* |  |  |  |  |  |
| A1N10HSH40 | *72374* | *15716* | *9355* |  |  |  |  |  |
| A1N10HSH70 | *55263* | *14417* | *8996* |  |  |  |  |  |
| A1N15HSH40 | *58605* | *14678* | *9069* |  |  |  |  |  |
| A1N15HSH70 | *44404* | *13411* | *8685* |  |  |  |  |  |
| A1N20HSH40 | *50335* | *13982* | *8865* |  |  |  |  |  |
| A1N20HSH70 | *38660* | *12845* | *8502* |  |  |  |  |  |
| A1N10OPT40 | *46509* | *13710* | *8785* |  |  |  |  |  |
| A1N10OPT70 | *34789* | *12495* | *8387* |  |  |  |  |  |
| A1N15OPT40 | *37394* | *12794* | *8489* |  |  |  |  |  |
| A1N15OPT70 | *28488* | *11684* | *8096* |  |  |  |  |  |
| A1N20OPT40 | *32031* | *12179* | *8279* |  |  |  |  |  |
| A1N20OPT70 | *25004* | *11184* | *7903* |  |  |  |  |  |
| A3N10RND40 | *168518* | *29520* | *4519* | 215567 | 30525 | 4591 | 68069 | 12051 |
| A3N10RND70 | *144410* | *28959* | *4477* |  |  |  |  |  |
| A3N15RND40 | *151298* | *29146* | *4492* |  |  |  |  |  |
| A3N15RND70 | *123329* | *28463* | *4439* |  |  |  |  |  |
| A3N20RND40 | *136267* | *28775* | *4463* |  |  |  |  |  |
| A3N20RND70 | *107172* | *28014* | *4401* |  |  |  |  |  |
| A3N10HSH40 | *112192* | *28294* | *4430* |  |  |  |  |  |
| A3N10HSH70 | *89160* | *27633* | *4374* |  |  |  |  |  |
| A3N15HSH40 | *93562* | *27772* | *4386* |  |  |  |  |  |
| A3N15HSH70 | *72246* | *27099* | *4325* |  |  |  |  |  |
| A3N20HSH40 | *80995* | *27397* | *4353* |  |  |  |  |  |
| A3N20HSH70 | *63239* | *26789* | *4296* |  |  |  |  |  |
| A3N10OPT40 | *75439* | *27254* | *4340* |  |  |  |  |  |
| A3N10OPT70 | *57830* | *26596* | *4277* |  |  |  |  |  |
| A3N15OPT40 | *61598* | *26758* | *4293* |  |  |  |  |  |
| A3N15OPT70 | *47076* | *26153* | *4231* |  |  |  |  |  |
| A3N20OPT40 | *53506* | *26430* | *4260* |  |  |  |  |  |
| A3N20OPT70 | *40867* | *25853* | *4198* |  |  |  |  |  |

*Supplementary Table S8: Cost per system per year*

| **Year** | **Cost per system / year (M€)** | | |
| --- | --- | --- | --- |
|  | **10 systems** | **15 systems** | **20 sys** |
| 2027 | 68.09 | 66.04 | 65.02 |
| 2028 | 29.49 | 28.03 | 27.29 |
| 2029 | 30.3 | 28.78 | 28 |
| 2030 | 31.13 | 29.55 | 28.74 |
| 2031 | 31.99 | 30.35 | 29.51 |
| 2032 | 40.59 | 38.69 | 37.71 |
| 2033 | 33.81 | 32.04 | 31.12 |
| 2034 | 34.77 | 32.93 | 31.98 |
| 2035 | 35.76 | 33.85 | 32.86 |
| 2036 | 35.95 | 34.09 | 33.16 |

**Supplementary References**

1. Lebreton, L., et al. (2018). Evidence that the Great Pacific Garbage Patch is rapidly accumulating plastic. Sci. Rep., 8. <https://doi.org/10.1038/s41598-018-22939-w>
2. Klink, D., Peytavin, A., & Lebreton, L. (2022). Size-dependent transport of floating plastics modeled in the global ocean. Front. Mar. Sci., 9, 1–9. <https://doi.org/10.3389/fmars.2022.903134>
3. Copernicus Marine Service (CMEMS). *Global Ocean Reanalysis Products (GLORYS12V1)*. Produced by Mercator Ocean. Accessed from: https://marine.copernicus.eu
   DOI: 10.48670/moi-00021
4. **Copernicus Climate Change Service (C3S)** (2017). ERA5: Fifth generation of ECMWF atmospheric reanalyses of the global climate. Copernicus Climate Change Service Climate Data Store (CDS). Available at: [https://cds.climate.copernicus.eu](https://cds.climate.copernicus.eu/)
5. Den Hertog, D., Pauphilet, J., Pham, Y., Sainte-Rose, B., & Song, B. (2024). Optimizing the path towards plastic-free oceans. Oper. Research, 0(0), ahead of print. <https://doi.org/10.1287/opre.2023.0515>
6. Charalambous, C. (2015). On the evolution of particle fragmentation with applications to planetary surfaces. *PhD Thesis,* Imperial College, London.
7. Song, Y. K., Hong, S. H., Jang, M., Han, G. M., Jung, S. W., & Shim, W. J. (2017). Combined effects of UV exposure duration and mechanical abrasion on microplastic fragmentation by polymer type. *Environ. Sci. Technol.*, 51(8), 4368–4376. <https://doi.org/10.1021/acs.est.6b06155>
